# The cytonuclear interactions during grapevine domestication

**DOI:** 10.1111/jipb.13968

**Published:** 2025-07-29

**Authors:** Ting Hou, Yanshuai Xu, Yang Dong, Jin Yao, Tianhao Zhang, Lianzhu Zhou, Xiangnian Su, Yi Zhang, Yingchun Zhang, Cheng Chen, Xiaoya Shi, Yuting Liu, Jiacui Li, Mengrui Du, Xinyue Fang, Sheng Yan, Sifan Yang, Wenrui Wang, Zhuyifu Chen, Siqi Qiao, Bilal Ahmad, Xiaodong Xu, Yanling Peng, Hua Xiao, Zhongxin Jin, Xiangpeng Leng, Cong Tan, Ling Tian, Chaochao Li, Yongfeng Zhou

**Affiliations:** ^1^ Institute of Grape Science and Engineering, College of Horticulture Qingdao Agricultural University Qingdao 266109 China; ^2^ State Key Laboratory of Tropical Crop Breeding, Tropical Crops Genetic Resources Institute Chinese Academy of Tropical Agricultural Sciences Haikou 571101 China; ^3^ Hunan Grape Engineering Technology Research Center, College of Horticulture Hunan Agricultural University Changsha 410128 China; ^4^ School of Management Shenzhen Polytechnic University Shenzhen 518000 China; ^5^ National Key Laboratory of Tropical Crop Breeding, Shenzhen Branch, Guangdong Laboratory of Lingnan Modern Agriculture, Key Laboratory of Synthetic Biology Ministry of Agriculture and Rural Affairs, Agricultural Genomics Institute at Shenzhen, Chinese Academy of Agricultural Sciences Shenzhen 518000 China; ^6^ National Key Laboratory for Tropical Crop Breeding, Hainan Key Laboratory for Biosafety Monitoring and Molecular Breeding in Off‐Season Reproduction Regions, Key Laboratory of Biology and Genetic Resources of Tropical Crops, Institute of Tropical Bioscience and Biotechnology, Sanya Research Institute, Coconut Research Institute Chinese Academy of Tropical Agricultural Sciences Haikou 571101 China; ^7^ BGI Research Shenzhen 518083 China

**Keywords:** grapevine, mitochondrial structural variation, NUMTs, NUPTs, viticulture

## Abstract

DNAs from the cytoplasmic genomes often communicate with the nuclear genome during regulation, development, and evolution. However, the dynamics of cytonuclear interaction during crop domestication have still been rarely investigated. Here, we examine cytonuclear interactions during grapevine domestication using pan‐mitogenome, pan‐plastome, and haplotype‐resolved nuclear genomes, all assembled from long‐read sequences across 33 wild and domesticated grapevine accessions. Structural variation shaped the mitogenomic variation in gene contents, leading to duplications of three specific genes during grapevine domestication (one *cox* and two *rpl* genes). Extensive genomic signals of cytonuclear interactions were detected, including a total of 212–431 nuclear–mitochondrial segments (NUMTs) and 95–205 nuclear–plastid segments (NUPTs). These results showed that NUMTs were under strong selection and were more abundant in cultivated grapes, whereas NUPTs dominated in wild grapes, indicating the evolutionary trajectories of cytonuclear interactions during grape domestication. Through Genome‐Wide Association Study (GWAS), we identified 84 candidate genes associated with mitochondrial–nuclear genome interactions. Among these, the *PFD1* gene acts as a signaling regulator, modulating specific signaling pathways regulated by the mitochondria. Interestingly, there are significantly more cytonuclear interaction genes near NUMTs than in other genomic regions, suggesting NUMT‐mediated interactions between the nuclear and mitochondrial genomes. Overall, our study provides evidence that NUMTs promote cytonuclear interaction during grapevine domestication, offering new insight into the impact of cytonuclear interactions on plant evolution, genetics, and breeding.

## Graphical abstract

Analysis of the grapevine pan‐mitochondrial genome reveals extensive structural variation, and shows that nuclear‐mitochondrial segments promote cytonuclear interaction during grapevine domestication, offering insights into the effect of cytonuclear interactions on plant evolution, genetics, and breeding.

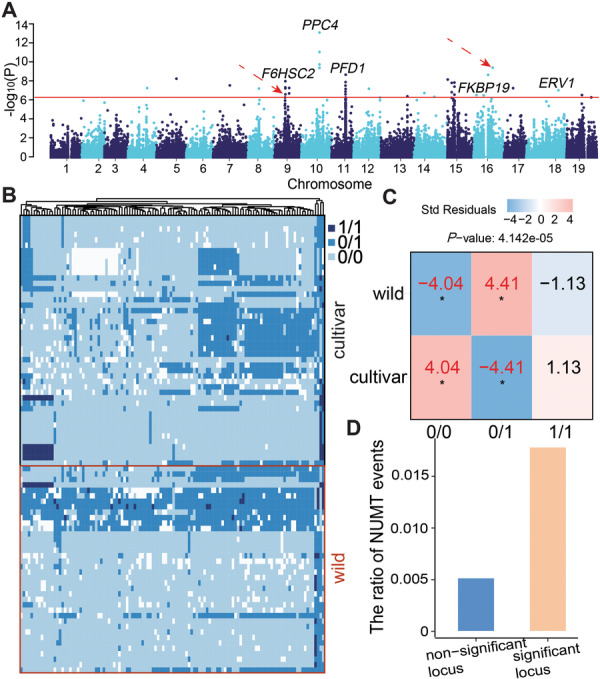

## INTRODUCTION

In plants, there has been a substantial and ongoing exchange of genetic material between the endosymbiotic‐originated organellar genomes (mitochondria and chloroplasts) and the nuclear genome (Timmis et al., 2004; Noutsos et al., 2005; Kleine et al., 2009; Keeling, 2010; Roger et al., 2017; Zhang et al., 2023, 2024). This dynamic interplay has led to the transfer of organellar DNA segments into the nuclear genome, resulting in the formation of nuclear–mitochondrial segments (NUMTs) and nuclear–plastid segments (NUPTs) (Leister, 2005). A substantial amount of organellar DNA has been found in the nuclear genome of flowering plants (Adams et al., 2000, 2001). Notable examples include the 620 kb NUMT in Arabidopsis (Lin et al., 1999; Stupar et al., 2001) and the 131 kb NUPT in rice (Consortium, 2003), which are the two largest organellar DNA insertion fragments reported to date. While the identification of human NUMTs has reached a relatively mature stage (Dayama et al., 2020), the identification of plant NUMTs remains a challenge. Traditional approaches rely primarily on sequence alignment strategies, such as using tools like BLAST to compare nuclear genome sequences with mitochondrial or plastid genome sequences. However, these methods are subject to homologous sequence interference (Richly and Leister, 2004; Noutsos et al., 2005; Michalovova et al., 2013), which can lead to incomplete identifications and numerous errors. Recent advances in sequencing technology, particularly the availability of long‐read sequencing technologies such as PacBio and Nanopore, have provided longer read lengths and higher accuracy, effectively overcoming the limitations of short‐read sequencing. As a result, the identification of NUMTs and NUPTs using PacBio HiFi sequencing technology has proven to be the most appropriate and efficient method for understanding their formation and evolutionary mechanisms.

Nuclear–cytoplasmic interaction is essential for the proper functioning of plant cells and plays a crucial role in plant evolution by maintaining the coordination between the nuclear and organellar genomes, thereby ensuring metabolic balance and genetic stability (Woodson and Chory, 2008). In plants, cytoplasmic male sterility (CMS), a condition characterized by pollen infertility, results from perturbations in the complex interplay between the mitochondrial and plastid genome and the nuclear genome. However, nuclear‐encoded restorer of fertility (Rf) genes can effectively suppress or overcome this sterility phenotype, demonstrating their co‐evolutionary relationship with CMS (Chase, 2007; Caruso et al., 2012). In several crops, including citrus, pepper, rice, and maize, the interaction between the *PPR* gene family and the mitochondrial genome has driven the evolution of CMS and fertility recovery (Kim et al., 2007; Toriyama, 2021; Wang et al., 2022; Yang et al., 2022). Furthermore, incompatibility between the nuclear and cytoplasmic genomes can lead to hybrid breakdown, as normal cellular function requires harmonious interactions between these two components (Leon et al., 1998; Levin, 2003). In the genus *Pisum*, interspecific hybrids between wild *Pisum sativum* ssp. *elatius* and the cultivated variety Sprint‐1 exhibit leaf development abnormalities and pollen sterility, mainly due to CMS resulting from nuclear–cytoplasmic conflicts (Bogdanova and Galieva, 2009). In *Rhododendron*, intersubgeneric crosses between evergreen azaleas (EV) and yellow‐flowered deciduous azaleas (JP) can result in genome–plastome incompatibility, particularly when EV plants are used as maternal parents. This incompatibility can result in albino traits in hybrid offspring due to the mismatch between the EV plastids and the JP nuclear genome (Ureshino et al., 2010). Nuclear–cytoplasmic interactions are pivotal in the mechanisms of reproductive isolation, genomic co‐adaptation, and the emergence of new species (Burton and Barreto, 2012; Barnard‐Kubow et al., 2016; Sloan et al., 2017), as evidenced by their role in creating barriers that restrict gene flow, sometimes leading to speciation (Baack et al., 2015), and their implications in the evolution of reproductive isolation, as highlighted by the varied evolutionary forces, such as post‐pollination barriers and genomic conflicts, that shape plant reproductive systems (Aalto et al., 2013). For example, in *Petunia*, the nuclear‐encoded RNA editing enzyme is unable to edit nucleotides in the tobacco plastid *atpA* gene, leading to the production of albino hybrids. In contrast, backcross hybrids exhibit normal phenotypes, underlining the critical role of genomic interactions in maintaining hybrid adaptability (Schmitz‐Linneweber et al., 2005).

As a source of genetic variation, NUMTs can also affect gene function, genome structure, and genetic diversity during domestication (Purugganan and Fuller, 2009). During the domestication of rice (*Oryza sativa*), NUMTs are shown to affect the expression of key domestication genes. For instance, mutations in the *SOP10* gene have been associated with reduced levels of reactive oxygen species (ROS), allowing rice to exhibit greater resistance to cold‐induced bleaching (Zu et al., 2023). While NUMTs occasionally have a positive effect on domestication, they can also present challenges. Research indicates that NUMTs may disrupt the coordination between mitochondrial and nuclear genes, leading to phenotypic abnormalities. For example, in Arabidopsis, the mismatched nucleocytoplasmic combination of Cvi‐0 and Sha can impair mitochondrial function and may be linked to metabolic disorders. This highlights the complex interactions between mitochondrial and nuclear DNA in plants (Roux et al., 2016). In addition to their influence on gene function, NUMTs and NUPTs can also affect haplotype structure. NUMTs/NUPTs accumulate gradually during polyploid evolution in *Triticum/Aegilops*, and these dynamic accumulation processes can affect haplotype structure (Zhang et al., 2024). Although studies on the effects of NUMTs/NUPTs on haplotypes in plants are limited, this study aims to investigate their potential impact on haplotype structure in grapevine accessions, thereby contributing to a deeper understanding of their role in plant domestication.

Grapes are widely cultivated and consumed worldwide as an important horticultural crop (Zhou et al., 2017; Grassi and De Lorenzis, 2021). Significant progress has been made in the study of grapevine and its domestication history, with well established genetic pathways (Zhou et al., 2017, 2019; Dong et al., 2023; Xiao et al., 2023). A substantial amount of high‐quality genomic and resequencing data has been published in grapevine (Shi et al., 2023; Peng et al., 2025), including pan‐genome studies (Liu et al., 2024; Guo et al., 2025), which capture the full spectrum of genetic diversity across cultivars. Expanding beyond the nuclear genome, recent advances in pan‐mitogenome and pan‐plastome research (Wang et al., 2022; Magdy et al., 2019) have demonstrated how organellar genome variation can influence traits and domestication. These comprehensive genomic resources, combined with grapevine's well‐characterized domestication history, make grapevine an ideal model system for investigating the impact of NUMTs and NUPTs on crop domestication (Xiao et al., 2025). The purpose of this study is to investigate the following three questions. First, how common are SVs in the mitochondrial genome of *Vitis*? Second, what are the distribution characteristics of NUMTs/NUPTs in the grape genome and are there any differences between NUMTs and NUPTs? Finally, do NUMTs affect the domestication and cytonuclear interactions of grapes? In this study, we constructed pan‐mitogenome and pan‐chlorogenome based on 33 grape samples and found that three genes (*infA*, *rpl14*, and *rpl36*) were lost in the East‐Asian wild grapevine mitogenomes. A *Vitis*‐specific structural variation (SV) in selective sweeps was identified based on the assembled mitochondrial genomes and population data across 100 grapevine genomes. In addition, we accurately identified NUMT and NUPT phenomena in 13 grapevine accessions and detected 84 candidate genes significantly associated with cytonuclear interactions through Genome‐Wide Association Study (GWAS) analysis.

## RESULTS

### The pan‐mitogenome and pan‐chlorogenome for grapevine

**Figure 1:**
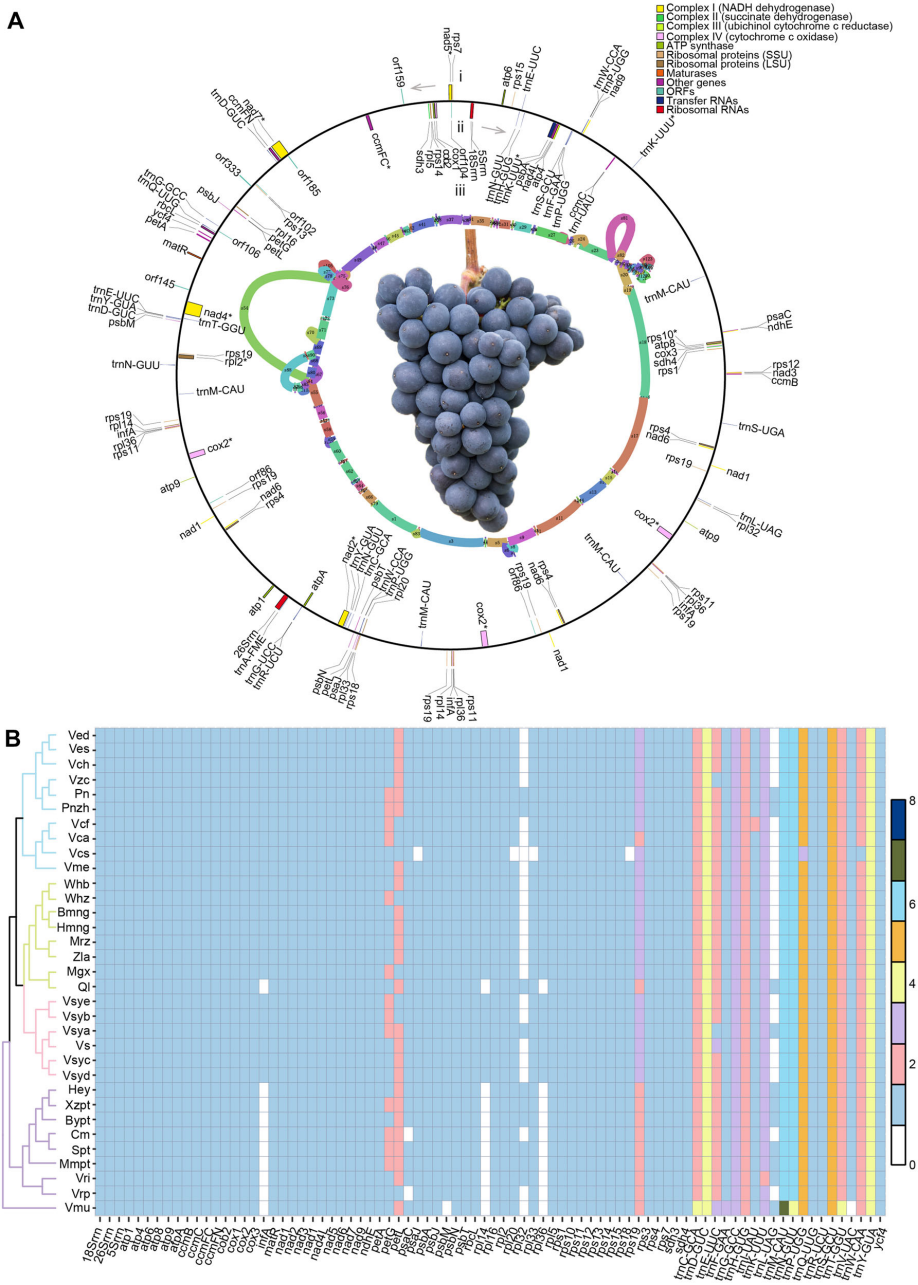
Pan‐mitogenome and structural variation in grapevine populations **(A)** The circular representation of grapevine pan‐mitogenome. The conserved gene structure was annotated for: (i) the forward strand, and (ii) the reverse strand; and the (iii) mitochondrial pan‐genome is indicated with a schematic drawing. **(B)** Phylogenetic tree (left panel) and gene content (right panel) of 33 *Vitis* accessions. Different colors represent the number of genes present in each mitogenome: Ved, *Vitis vinifera*; Ves, *Vitis vinifera*; Vch, *Vitis vinifera* cv. Chardonnay; Vzc, *Vitis vinifera* cv. Zinfandel; Pn, *Vitis vinifera*—PN40024; Pnzh, *Vitis vinifera* cv. Pinot Noir; Vcf, *Vitis vinifera* cv. Cabernet Franc; Vca, *Vitis vinifera* cv. Carmenere; Vcs, *Vitis vinifera* cv. Cabernet Sauvignon; Vme, *Vitis vinifera* cv. Merlot; Whb, *Vitis vinifera* “Thompson Seedless”; Whz, *Vitis vinifera* “Black Monukka”; Bmng, *Vitis vinifera*; Hmng, *Vitis vinifera*; Mrz, *Vitis vinifera* “Manicure Finger”; Zla, *Vitis vinifera* “Julian”; Mgx, *Vitis vinifera* “Muscat Hamburg”; Ql, *Vitis labrusca* *×* *Vitis vinifera*; Vsye, *Vitis vinifera* ssp. *sylvestris*; Vsyb, *Vitis vinifera* ssp. *sylvestris*; Vsya, *Vitis vinifera* ssp. *sylvestris*; Vs, *Vitis vinifera* ssp. *sylvestris*; Vsyc, *Vitis vinifera* ssp. *sylvestris*; Vsyd, *Vitis vinifera* ssp. *sylvestris*; Hey, *Vitis heyneana*; Xzpt, *Vitis adenoclada*; Bypt, *Vitis piasezkii*; Cm, *Vitis davidii*; Spt, *Vitis amurensis*; Mmpt, *Vitis retordii*; Vri, *Vitis riparia*; Vrp, *Vitis rupestris*; Vmu, *Vitis rotundifolia*.

We utilized 33 long‐read sequencing data samples to assemble mitogenomes and plastid genomes from four populations (wine, table, *Vitis vinifera* ssp. *sylvestris* (Syl), *Vitis* wild relatives (Vwr); Table S1), resulting in a pan‐mitogenome (Grapepan_mt v.1.0, 1.28 Mb) (Figure 1A) and a pan‐chlorogenome (Grapepan_cp v.1.0, 138 kb) (Figures S1, S2). The pan‐mitogenome is about twice the size of a single mitochondrial genome, and contains 144 continuous segments from the Bmng mitochondrial genome, which serves as the reference genome in this study. The pan‐chlorogenome, about the same size as a single plastid genome, contains 31 continuous segments. The assembled mitochondrial genomes ranged in size from 663.16 kb (*Vitis rotundifolia*: Vmu) to 817.37 kb (*Vitis vinifera* cv. Carmenere: Vca) and contained 108–129 genes. Reverse repeats were rare in most Vwr grapes, while forward repeats were more common (Table 1). The GC content of the mitochondrial genomes has remained relatively consistent, with an average of approximately 44%. The proportion of TEs in the mitochondrial genome is about 22% (Tables S2, S3). For plastid genome assembly, all 33 grape genomes were successfully assembled into the typical four‐part ring structure, ranging in size from 159,011 bp (*Vitis vinifera* ssp. *sylvestris*: Vs) to 163,619 bp (*Vitis vinifera* cv. Cabernet Franc: Vcf). The average GC content of the plastid genomes was 37.38%, while TEs accounted for about 87% (Tables S4, S5).

**Table 1:** Summary of the 33 mitochondrial assemblies

Species name	Sample	Group	Genome size (bp)	Gap	Number of genes	Number of repeat sequences		
						F	P	R
*Vitis vinifera*	Ved	Wine	817,057	1	116	27	21	2
*Vitis vinifera*	Ves	Wine	817,062	1	116	26	22	2
*Vitis vinifera* cv. Chardonnay	Vch	Wine	816,446	0	116	25	21	4
*Vitis vinifera* cv. Zinfandel	Vzc	Wine	809,242	0	115	25	20	5
*Vitis vinifera* cv. PN40024	Pn	Wine	773,298	0	119	23	23	4
*Vitis vinifera* cv. Pinot Noir	Pnzh	Wine	773,300	0	119	24	22	4
*Vitis vinifera* cv. Cabernet Franc	Vcf	Wine	816,200	2	117	27	21	2
*Vitis vinifera* cv. Carmenere	Vca	Wine	817,368	1	115	27	21	2
*Vitis vinifera* cv. Cabernet Sauvignon	Vcs	Wine	810,760	1	108	30	20	0
*Vitis vinifera* cv. Merlot	Vme	Wine	816,568	0	116	25	20	5
*Vitis vinifera* cv. Thompson Seedless	Whb	Table	816,566	0	116	25	20	5
*Vitis vinifera* cv. Black Monukka	Whz	Table	817,063	1	116	27	21	2
*Vitis vinifera*	Bmng	Table	816,336	0	116	26	19	5
*Vitis vinifera*	Hmng	Table	816,566	0	116	25	20	5
*Vitis vinifera* cv. Manicure Finger	Mrz	Table	816,565	0	116	25	20	5
*Vitis vinifera* cv. Julian	Zla	Table	816,565	0	116	25	20	5
*Vitis vinifera* cv. Muscat Hamburg	Mgx	Table	817,057	1	116	29	19	2
*Vitis labrusca* *×* *Vitis vinifera*	Ql	Table	755,036	0	114	26	24	0
*Vitis vinifera* ssp. *sylvestris*	Vsye	Syl	817,077	1	116	27	21	2
*Vitis vinifera* ssp. *sylvestris*	Vsyb	Syl	817,072	1	116	26	21	3
*Vitis vinifera* ssp. *sylvestris*	Vsya	Syl	773,074	0	118	22	22	6
*Vitis vinifera* ssp. *sylvestris*	Vs	Syl	790,627	0	117	26	19	5
*Vitis vinifera* ssp. *sylvestris*	Vsyc	Syl	816,500	0	116	28	22	0
*Vitis vinifera* ssp. *sylvestris*	Vsyd	Syl	816,463	0	116	28	22	0
*Vitis vinifera* ssp. *sylvestris*	Hey	Vwr	757,300	0	114	30	20	0
*Vitis adenoclada*	Xzpt	Vwr	784,133	0	115	29	21	0
*Vitis piasezkii*	Bypt	Vwr	791,319	1	114	32	18	0
*Vitis davidii*	Cm	Vwr	738,535	1	112	31	19	0
*Vitis amurensis*	Spt	Vwr	774,208	0	115	29	21	0
*Vitis retordii*	Mmpt	Vwr	762,981	0	115	29	21	0
*Vitis riparia*	Vri	Vwr	755,294	0	113	27	23	0
*Vitis rupestris*	Vrp	Vwr	795,535	5	110	39	11	0
*Vitis rotundifolia*	Vmu	Vwr	663,157	0	117	33	17	0

F, P and R in the table indicate forward repeats, palindromic repeats, and reverse repeats, respectively.

To study the SV and its impact on cytoplasmic genes, we further performed gene copy number analysis and detected a total of 55 protein‐coding genes that were universally present in all 33 grape mitochondrial genomes. Compared with domesticated grapes, four protein‐coding genes (*infA*, *rpl14*, *rpl36* and *rps19*) were lost in nine wild grapes and one cultivated grape (Figure 1B). In addition, other genes such as *psaC*, *psaJ*, *rpl2*, *rpl33*, *rps18*, *psbM* and *rpl32* were lost in various grape species. The replication of the *petL* gene occurred in almost all grapes, while the replication of the *rps19* gene was observed in all grapes. For the plastid genome, most protein‐coding genes are single copies. Unlike the mitochondrial genome, the plastid genome has a maximum copy number of two genes, among which five genes, *ndhB*, *pafI*, *rpl23*, *rps7* and *ycf2*, each have two copies in the 33 grape genomes (Figure S3). In addition, we selected 13 representative grapes to observe the rearrangement phenomenon among different grape mitochondrial genomes and revealed rearrangements among different grape mitochondrial genomes. Specifically, more rearrangement events took place in the Vwr group than in the other groups, while fewer rearrangement events were observed within the table grape group, ranging from two to six. Although the Vwr population had a greater number of rearrangement events, the mitochondrial genome structure of *Vitis heyneana*, *Vitis piasezkii*, and *Vitis retordii* was similar. Throughout the evolution of grapes, the Vwr population experienced more genetic rearrangement events than the other three populations (Figure S4).

### SV and artificial selection on mitogenomes during grape domestication

**Figure 2:**
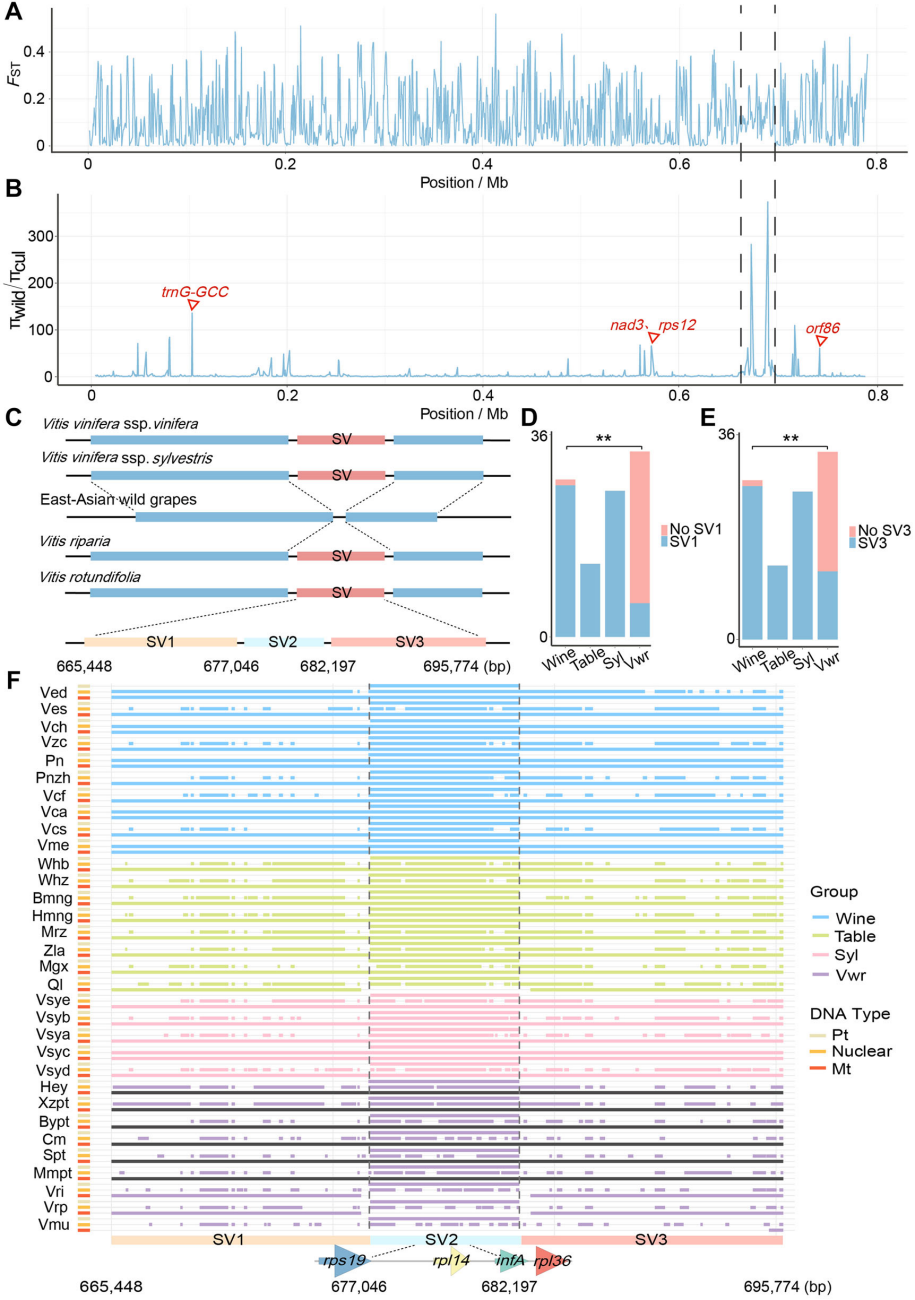
SV analysis of the mitogenomes of grape **(A)** Distribution of *F*_ST_ values in four grape populations. **(B)** Distribution of π_wild_/π_cul_ ratios; cul means cultivated grape population; dotted regions represent the SV (from 665,448 to 682,197 bp) in East‐Asian wild grapes. **(C)** A SV was identified in East‐Asian wild grapes (*V. heyneana, V. adenoclada, V. piasezkii, V. davidii, V. amurensis, V. retordii*). The SV sequence is further divided into three parts (SV1, SV2, and SV3). **(D**, **E)** Appearance times of SV1 **(D)** and SV3 **(E)** in the four grape groups. Syl is *Vitis vinifera* ssp. *sylvestris* and Vwr is *Vitis* wild relatives. Red represents the samples not containing the SV, and blue represents the samples containing the SV. **(F)** Homologous sequences of SV1, SV2, and SV3 in 32 mitogenomes, plastid genomes and nuclear genomes. The gray bar indicates no information was available.

To examine the potential impact of selective sweeps on mitochondrial genetic diversity, we conducted selective sweeps scans using resequencing data from 100 grapevine samples, representing four distinct populations: 13 from table, 28 from wine, 26 from Syl, and 33 from Vwr grapevine (Table S6). Our analysis identified 5.82% (46.00 kb out of 790.627 kb) of the mitochondrial genome under selection, which included three protein‐coding genes (*nad3*, *rps12*, *ccmFC*), one tRNA, and one *orf* gene (Figure 2A, B; Table S7). Within these selected regions, we identified a ~30‐kb deletion (SV: 665.448–695.774 kb) among six East‐Asian wild grapes (Hey, Xzpt, Bypt, Cm, Spt, Mmpt), which belong to the Vwr species (Figure 2C). This SV was further divided into three parts (SV1, SV2, and SV3). SV1 and SV3 were mitochondria‐specific sequences, while SV2 shared high homology (over 99% BLASTN) with a plastid genome sequence (Table S8). Thus, our analysis focused on the frequencies of SV1 and SV3 across table, wine, Syl, and Vwr grapevines. Our results showed that 96.43% (27/28) of wine grapes contained SV1 at a significantly higher frequency compared to Vwr grapes (18.18%) (Figure 2D) (chi‐squared test, *P*‐value = 4.814474e‐09). Similarly, SV3 was also more prevalent in wine grapes than in Vwr (chi‐square test, *P*‐value = 4.209265e‐06) (Figure 2E).

Among the *Vitis* mitogenomes, large sequence fragments of the SV first appeared in Vmu grape and subsequently spread to domesticated grapes (Figure 2F). Notably, all cultivated grape mitochondrial genomes possessed large SV fragments. Regarding nuclear sequence matching, most wine grapes (except for Vcs and Vcf) shared over 50% of the SV sequences with their nuclear genomes, with proportions of 46.38% and 48.38%, respectively. Syl and Vwr grapes exhibited a broader range of sequence sharing, ranging from 33% to 100%, whereas table grapes displayed a narrower range, from 47.46% to 65.57% (Table S9). These results indicate that SV changed repeatedly and continued to evolve during grape domestication.

### Asymmetric accumulations of NUMTs/NUPTs in grapevine nuclear genomes

**Figure 3:**
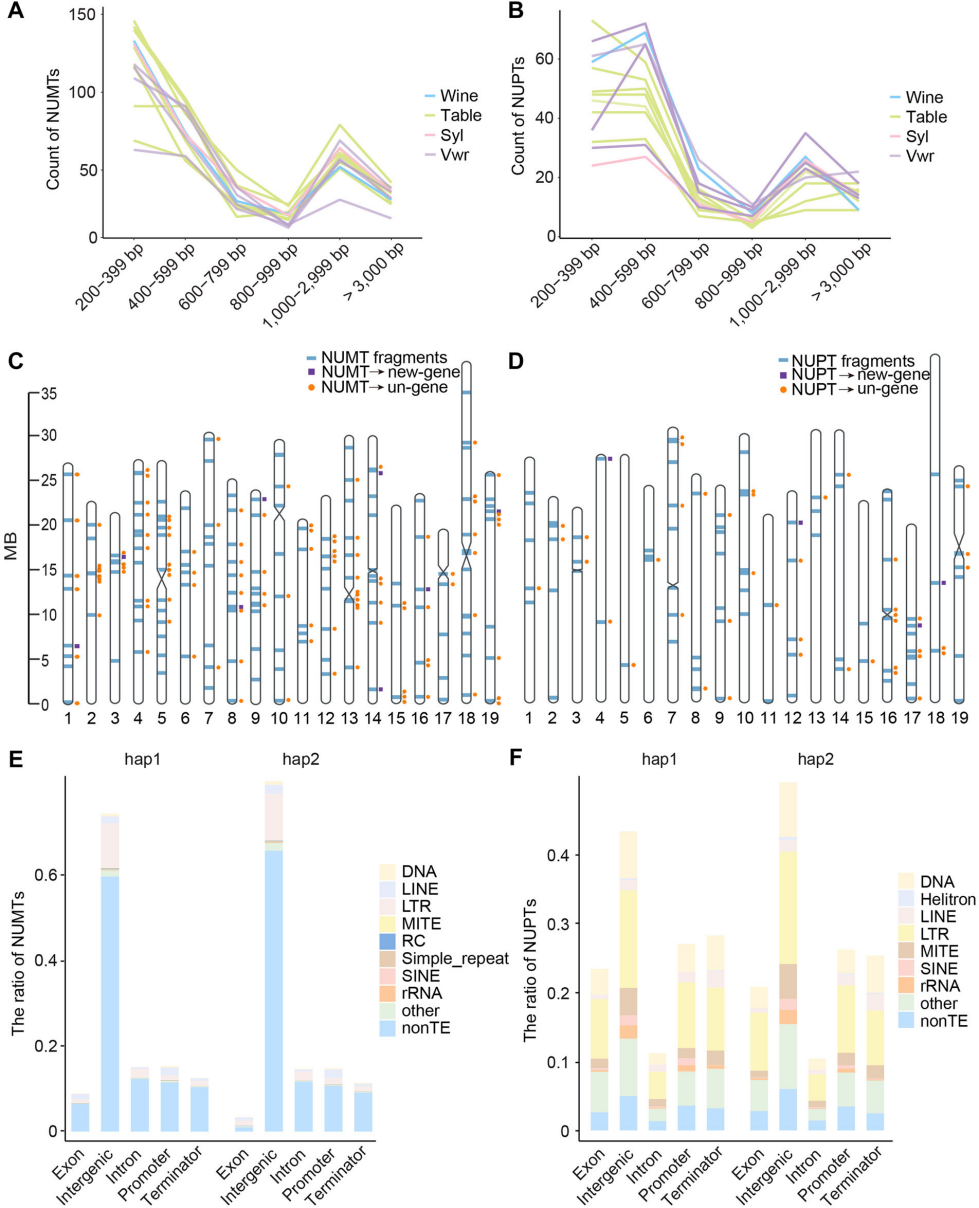
Characteristic of NUMTs and NUPTs in grapes **(A**, **B)** The length and number of NUMTs **(A)** and NUPTs **(B)** of four grape populations. The line chart shows the number distribution of NUMTs and NUPTs for different grape varieties at different length intervals. **(C**, **D)** The distribution of NUMTs **(C)** and NUPTs **(D)** in the nuclear chromosomes of Whzhap1. Blue indicates the location on nuclear chromosomes. The purple box indicates that the mitochondrial/plastid genome did not form genes before transfer to the nucleus, and formed new genes after transfer; The orange circle indicates that the mitochondrial/plastid genome was a gene before it was transferred to the nucleus and is not a gene after it is transferred. **(E**, **F)** Composition of NUMTs **(E)** and NUPTs **(F)**. The histogram shows the ratio distribution of NUMTs and NUPTs in transposable elements (TE) and non‐TE regions of different gene structures in the two haplotypes. Intergenic region: the region of the genome other than promoter, terminator, and gene regions; Promoter region: within 2 kb upstream of genes; Terminator region: within 2 kb downstream of genes.

To explore whether the four genes (*infA*, *rpl14*, *rpl36* and *rps19*) lost in East‐Asian wild grape mitochondria were transferred to NUMTs/NUPTs, and to examine the patterns of NUMTs/NUPTs during grapevine evolution, we successfully identified 212 to 431 high‐confidence NUMT and 95 to 205 NUPT fragments in 13 grape samples using PacBio HiFi data, following the application of a series of quality control filters (Figure S5; Tables S1, S10). First, the four genes (*infA*, *rpl14*, *rpl36* and *rps19*) were found in their NUPTs (Table S11). Additionally, the most abundant NUMTs were found in the range 200–399 bp, whereas NUPTs were predominantly found in the range 400–599 bp (Figure 3A, B). The number of NUMTs and NUPTs varied in different species. Cultivated grapes typically contained more NUMTs, whereas wild species tended to have a higher abundance of NUPTs (Figure S6). However, haplotype variation within species showed minimal differences in the numbers of NUMTs and NUPTs (Table S10). The distribution of NUMTs and NUPTs within the nuclear genome varied by species, chromosome location and gene. Some NUMTs were functional genes before being transferred to nuclear DNA, but lost their gene function after transfer, either due to mutations during the transfer process or because they are no longer needed in their new environment. Some NUMTs were not genes before transfer but formed neofunctionalized genes after integration into the nuclear genome. This suggests that these DNA fragments may have acquired new functions after being transferred. In most grapevine species, NUMTs were less abundant on chromosomes 3 and 17, while NUPTs were less frequent on chromosomes 2 and 18 (Figure 3C, D). Conversely, NUMTs were more abundant on chromosome 18, and NUPTs were more abundant on chromosome 17 (Figures S7–S10).

We further investigated the frequency and distribution of mitochondrial and plastid sequences transferred into the nuclear genome, finding the highest transfer rates in intergenic regions. NUMTs and NUPTs were differentially associated with transposable elements (TEs), with NUMTs being mostly non‐TE sequences and NUPTs having a higher proportion of TE‐associated sequences, including DNA, LINE, LTR, MITE, SINE, and rRNA elements (Figure 3E, F). Interestingly, in some cultivated grapes (e.g., Whb, Whz, Bmng), NUMTs in promoter regions were more frequently associated with LINE elements, while wild grapes (e.g., Hey, Bypt) showed a predominance of LTR elements. Regarding the distribution of NUMTs and NUPTs within the nuclear genome, there were generally few differences between haplotypes, except for Hey and Mrz, where Hey showed significant differences in the exon and intergenic regions, while Mrz exhibited more differences in the intergenic region (Figure S11). For NUPTs, the proportions of each region and TE were generally similar between hap1 and hap2, with LTR having the highest proportion. However, some cultivars, such as Whb, Whz, Mgx, and Ql, showed significant differences in the exon regions, and the differences in the proportions of Hey and Vs (wild grapes) across regions were more pronounced (Figure S12). These results highlight species‐specific and haplotype‐level variations in NUMTs and NUPTs accumulation, suggesting distinct evolutionary patterns shaping the transfer and retention of organelle‐derived sequences in grapevine genomes.

### Homozygous NUMTs drive the genomic evolution of grapevine

**Figure 4:**
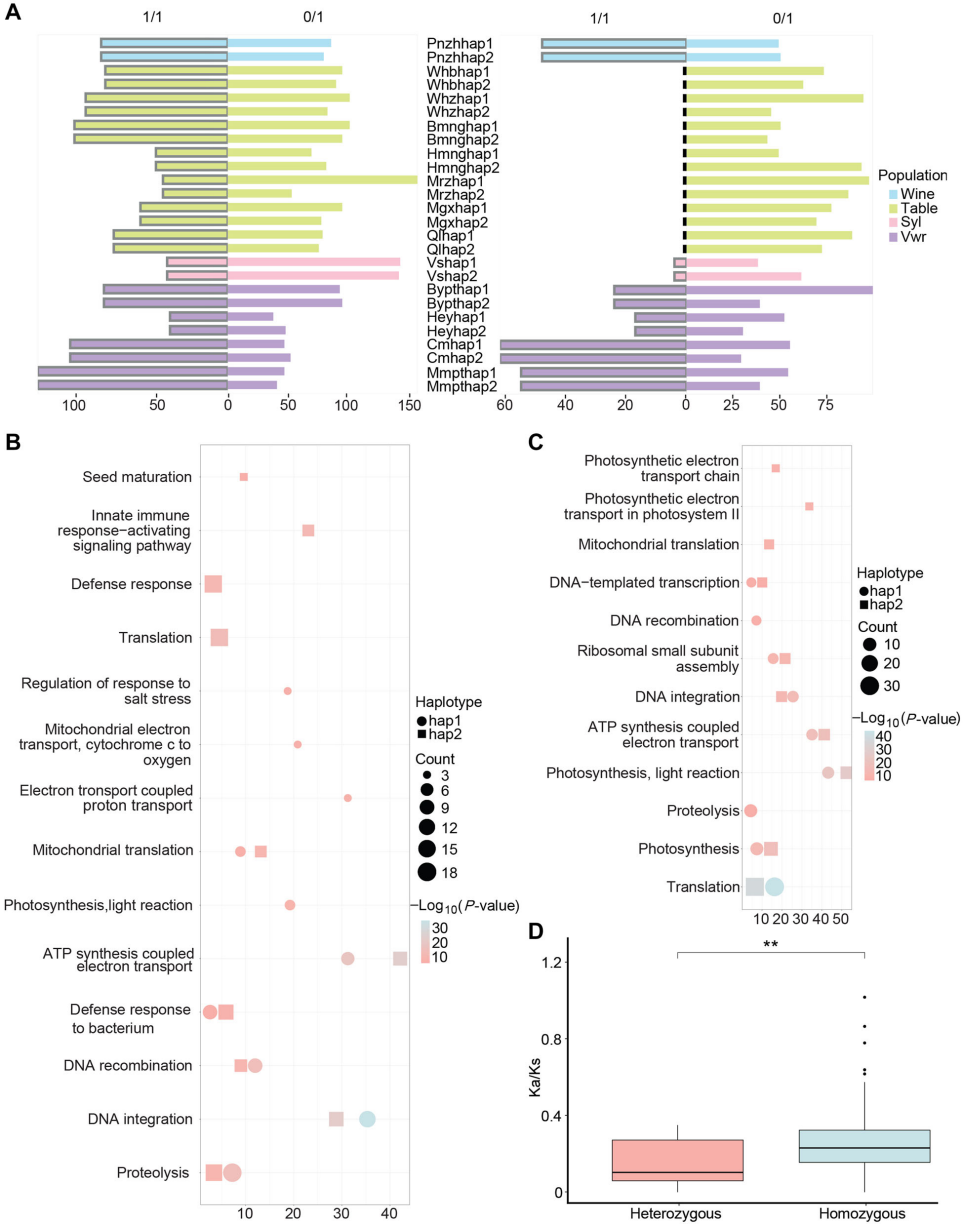
Comparative analysis of heterozygous and homozygous genes of NUMTs and NUPTs during the domestication of grapes **(A)** Frequency plots of NUMTs (left) and NUPTs (right) in both haplotypes of four grape groups. 0/1 = heterozygous, 1/1 = homozygous. **(B**, **C)** GO enrichment (BP, biological process) of hap1 and hap2 genes captured exon and promoter of NUMTs **(B)** and NUPTs **(C)**. In the diagram, the circles represent hap1, while the squares represent hap2. The size of the circles indicates the number of genes, with larger circles indicating a greater number of genes. **(D)** Ka/Ks of heterozygous and homozygous NUMTs genes in wild and cultivated grapes. Significant *P‐*value for difference: **P < 0.01.

To investigate the potential impact of homozygous and heterozygous NUMTs/NUPTs on grape evolution, we observed an interesting pattern: homozygous NUMTs were more prevalent in wild grapes, while heterozygous NUMTs, especially heterozygous NUPTs, became increasingly abundant in domesticated grapes (Figure 4A). In total, 834 genes were captured in hap1 and 775 genes in hap2 of NUMTs, of which 598 and 580 genes had UniProt IDs, respectively. For NUPTs, 763 genes were captured in hap1 and 559 in hap2, with 537 and 425 genes having UniProt IDs (Tables S12, S13). Furthermore, we predicted 352 deleterious variation sites in 119 genes. Interestingly, one *MIK2‐like* gene (*Vitvi031348*) plays a dual role in sensing the conserved SCOOP feature MOBS in plants and microorganisms, and triggering plant immunity (Hou et al., 2021; Stahl et al., 2022). This gene underwent NUMT and NUPT events in three table grape varieties, suggesting that defense‐related genes in the nuclear genome are more susceptible to “attacks” by mitochondrial and plastid gene fragments. The newly inserted NUMT and NUPT fragments into the *MIK2* gene increased harmful variations, which may adversely impact the adaptability of grapes (Tables S14, S15).

Functional annotation revealed that NUMTs were significantly enriched in biological processes related to defense responses, such as defense response to bacteria, plant‐type hypersensitive response, highlighting the critical role of these homozygous genes in grapevine survival and adaptation. We also discovered unique processes, such as the regulation of response to salt stress, are unique to certain haplotypes, which helps the organism adapt to and resist salt stress, thereby ensuring survival and normal growth. In contrast, heterozygous genes contributed to novel biological functions (Figures 4B, S13; Table S16). For NUPTs, processes like photosynthesis and photosynthetic electron transport in photosystem II were shared in both haplotypes, supporting basic metabolism. Notably, we observed that *Q0ZIY9* (associated with photosynthesis)—a gene expressed in cultivated grapes (Mgx) but absent in wild grapes (Cm)—may represent a domestication‐selected gene retained during grape evolution. Haplotype‐specific processes like proteolysis were found to be crucial for cell function and organism survival (Figures 4C, S14; Table S17).

Differential NUMT genes were significantly enriched in metabolic pathways, oxidative phosphorylation, and photosynthesis, all of which are equally important in both haplotypes, indicating their indispensability in grape physiology. However, aminoacyl‐tRNA biosynthesis was only significantly enriched in hap2 (Figure S15), enhancing protein synthesis efficiency in heterozygotes under specific conditions. Differential gene enrichment analysis of NUPTs revealed that several metabolic pathways, including photosynthesis and RNA polymerase activity, critical for grapevine metabolism, along with a unique pathway in carbon metabolism (Figure S16), which is central to plant life and involves energy conversion and substance synthesis. Furthermore, Ka/Ks ratio analysis showed significantly higher values for homozygous NUMT genes in wild grapes compared with cultivated grapes (Figure 4D). This marked difference suggested that homozygous genes have experienced stronger positive selection pressure and may have influenced the evolution of grapevine.

### NUMTs facilitate cytonuclear interactions in grapevines

**Figure 5:**
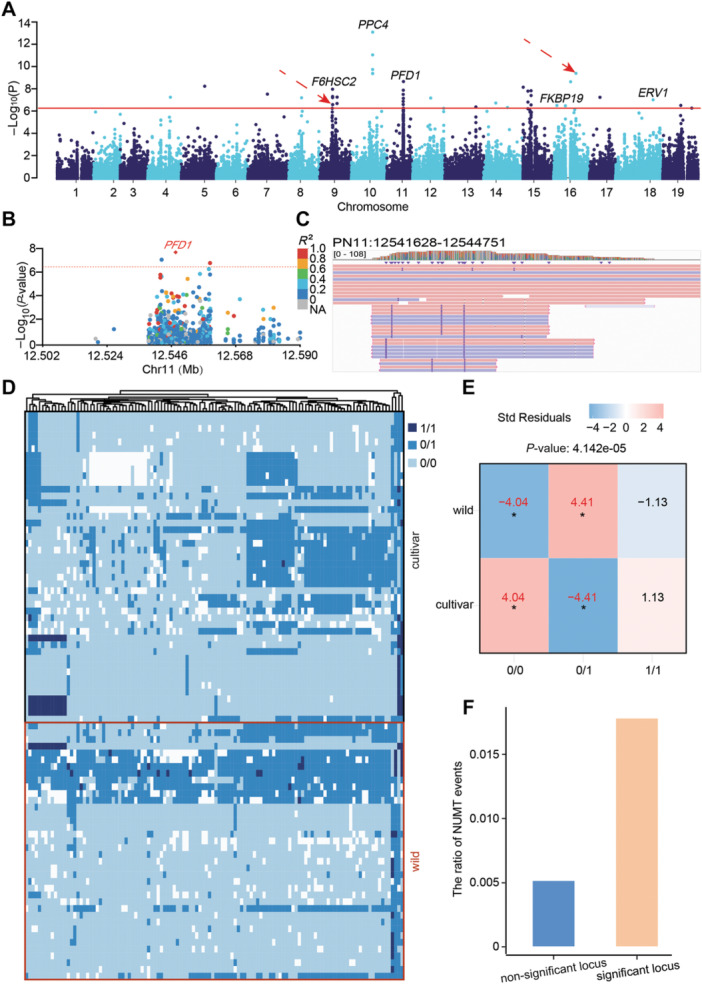
Genomic insights into mitonuclear interactions: candidate genes and variant analysis **(A)** Manhattan plot of GWAS analysis for mitochondrial single nucleotide polymorphism (SNPs) in grapevine. The horizontal line represents the significance threshold. The dotted red arrows indicate that a nuclear–mitochondrial segments (NUMT) event occurred at that site. **(B)** SNPs surpassing this threshold are highlighted in the zoomed‐in plot, with the most significant SNP marked by a diamond. **(C)** Depth map of the *Vitvi020593* gene and its surrounding sequences in IGV. The color differentiation is based on the read strand orientation, where pink signifies a match between the read direction and the gene's orientation, and purple indicates the opposite read direction and gene's orientation. **(D)** Heatmap of significant associated variation sites in the nucleus and mitochondria between wild and cultivated grapes. Colors represent allelic state: light blue = homozygous reference allele, dark blue = heterozygous, and ink blue = homozygous alternative allele. Each row illustrates the genotype of one sample. **(E)** Correlation analysis of nucleoplasmic significant association loci with genotype in wild and cultivated grapes. Significant *P*‐value for difference: *P < 0.05. **(F)** The ratio of NUMT events at nucleocytoplasmic significant association sites and non‐significant sites.

To reveal the potential co‐evolution between the grapevine cytoplasmic genome and nuclear genome, we adopted the approach described by Lian et al. (2024), using two populations (50 cultivated and 50 wild grapes; Table S6), and detected significant mitonuclear and plastonuclear association signals (Figures 5A, S17–S19). GO enrichment of 84 candidate genes from mitonuclear GWAS revealed their involvement in DNA integration, recombination, and biosynthesis (Figure S20). Similarly, 133 candidate genes from nuclear–plastid interactions were enriched in proteolysis, defense response and protein export from the nucleus (Figure S21). Among the candidate genes, *Prefoldin subunit 1* (*PFD1*, *Vitvi020593*), which is closely associated with mitochondrial variation, was identified on chromosome 11 of grapevine (Figure 5B). The PFD subunit, also known as the gene involved in microtubule synthesis (GIM), forms a heterohexameric PFD complex involved in the folding of microtubules and actin. Notably, the accumulation of PFDs is associated with the degradation of HY5, which plays a key role in low temperature response, suggesting that PFDs may be involved in the plant's response to abiotic stress (Perea‐Resa et al., 2017). Furthermore, the coverage of the *PFD1* gene in the Integrative Genomics Viewer (IGV v2.12.3) was significantly higher than the surrounding nuclear genome sequences, confirming its localization in the mitochondrial genome (Figure 5C).

The heatmap revealed a pattern in the distribution of heterozygous and homozygous sites, showing notable differences between cultivated and wild grapes at these loci. Cultivated grapes tended to have a higher frequency of homozygous sites at core‐mitochondrial interaction loci. This pattern may be attributed to the selection of beneficial genotypes during domestication in cultivated grapes. In contrast, wild grapes retained a higher proportion of heterozygous sites, likely contributing to greater genetic diversity and environmental adaptability (Figure 5D, E). Finally, we found that NUMT events were significantly enriched near significant nuclear and mitochondrial association sites compared to non‐significant association sites (Figure 5F), suggesting that NUMTs play a facilitating role in cytonuclear interaction and contribute to the co‐evolution of the grapevine cytoplasmic and nuclear genomes.

## DISCUSSION

The primary objectives of this study were to use grapevines as a model to explore cytoplasmic genome variation, patterns of cytonuclear interaction, and the impact of NUMTs and NUPTs on nuclear genome evolution. We successfully assembled the mitochondrial and plastid genomes of 33 grapevine samples and identified NUMTs and NUPTs. By examining their insertion modes in detail, we reveal their distribution patterns in the grape genome and propose possible mechanisms for their insertion. These findings provide new insight into the dynamics of cytonuclear transfer and confirm the role of NUMTs and NUPTs in grape domestication (Figures 3, 4). Genome‐wide association analysis further identified significant nuclear genes associated with mitochondrial and plastid variation, suggesting a complex signaling network in cytonuclear interaction. Additionally, nuclear mutations may influence the pattern of cytonuclear interactions. Notably, we observed differences in variable loci between wild and cultivated grapes, suggesting that certain loci may have facilitated cytonuclear co‐evolution. Although our study has yielded meaningful results, there are still areas for improvement. For instance, in the assembly of the mitochondrial genome, we focused primarily on the main circular structure, which limited our ability to fully explore the potential conformational diversity of mitochondria. This may have restricted a more comprehensive understanding of the genome's complexities. Meanwhile, the formation mechanisms of NUMTs/NUPTs remain unclear.

Plant mitochondrial genomes exhibit variable genome sizes and structures (Gualberto and Newton, 2017; Cole et al., 2018). Here, we assembled and annotated the mitochondrial genomes of 33 grapevine species, providing new insight into this variability. Previous reports have indicated that the larger size of grapevine mitochondrial DNA is not due to gene content but rather the expansion of its intergenic regions (Goremykin et al., 2009). We propose that copy number variations within the same clade may influence mitochondrial genome size, with larger genomes often being associated with lower copy numbers (Figure S22). This phenomenon may be because large insertions cannot be effectively corrected by homologous repair when the number of mitogenome copies is low. Plant mitochondrial genomes undergo significant structural changes during the co‐evolution of endosymbionts and hosts, leading to increased genomic complexity. Compared with the highly conserved plastid genome, the mitochondrial genome exhibits greater SV. In grapes, we observed significant rearrangements within mitochondrial genomes of the same genus, with a more than 10‐fold difference in rearrangement rates among 11 genera of the Rosaceae family (Sun et al., 2022), Moreover, several studies have shown that nuclear gene variants (such as *MSH1*) (Gualberto and Newton, 2017) can also contribute to mitochondrial genome rearrangements. The mitochondrial genome of the Ql, a cultivated variety, showed three gene loss events that are common in wild grapes. This change is likely to have been due to its breeding background, as it is a cross between an American species and a European subspecies. These complex structures pose challenges for accurate genome assembly, especially as most mitochondrial genomes are simplified to the main circular structure. As a result, many important structural features may be overlooked. Therefore, future studies should focus on the complete assembly of mitochondrial genomes to better reveal their complex structural characteristics and gain a deeper understanding of their role in plant evolution and function.

To better understand the role of NUMT and NUPT fragments in domestication, we examined their insertion patterns. Previous studies have shown that organelle DNA is preferentially inserted into centromeric regions, and then propagated via transposition (Michalovova et al., 2013). Nevertheless, our analysis revealed that, in some varieties, insertions did not occur in the centromeric regions (Tables S18, S19), possibly due to limitations in assembly quality. Similar to findings in *Sorghum* (Zhang et al., 2023), we observed an asymmetric deposition pattern for NUMTs and NUPTs in grapevines, with different distributions among cultivars and transfer regions. Despite these similar patterns, we found that the insertion rates of NUMTs and NUPTs were not constant. The number of NUMTs increased, while NUPTs decreased during grapevine domestication. This may be due partly to assembly quality or the potential benefits of transferring mitochondrial function genes to the nucleus for improved environmental adaptation or domestication selection. Additionally, no homozygous NUPTs were found in table grapes, suggesting stronger selective pressures on cultivated varieties (Figure 4A). Homozygous plastid insertions into the cultivated nuclear genome may lead to lethal effects or produce traits unfavorable for domestication and, thus, these insertions may have been eliminated by natural selection. Regarding NUMTs, we observed that their sequences retained a GC content similar to that of the source mitochondrial genome, but the GC content of these transferred sequences was lower than that of the surrounding nuclear genome, indicating that they have been less influenced by the nuclear genome during evolution and exhibit an independent evolutionary pattern. In contrast, NUPT sequences not only have a GC content similar to that of the source plastid genome but also resemble the surrounding nuclear genome, suggesting that these fragments are more structurally stable.

Cytonuclear interactions, which involve the cooperative interplay between nuclear and cytoplasmic‐encoded gene products, are essential for the maintenance of plant physiology (Sloan et al., 2018; Kan et al., 2024). These interactions are key to speciation and biological evolution, exerting significant influence on genome stability and function (Chase, 2007; Sharbrough et al., 2017; Zupok et al., 2021). For example, hybridization experiments in Helianthus species have shown that cytonuclear interactions between different ecotypes profoundly impact hybrid fitness (Lexer et al., 2003; Sambatti et al., 2008). Moreover, Patrick Chinnery's team discovered that some children had mitochondrial DNA “insertions” within their nuclear DNA, which were not present in their parents. These insertions were occasionally associated with rare diseases, highlighting the dynamic interplay between mitochondrial and nuclear DNA (Wei et al., 2022). Our research further supports this notion. Through GWAS in grapes, we identified genes (*RUN1* and *RPV1*) associated with disease resistance that are potentially located at the junction of the nuclear and mitochondrial genomes. Ka/Ks analysis of these genes revealed that they underwent functional selection after integration into the nuclear genome (Figure S23), consistent with Patrick Chinnery's findings. This suggests ongoing co‐evolution between the nuclear and mitochondrial genomes. In conclusion, cytonuclear interactions are important in biological evolution and speciation. Our findings further support this notion and provide new insight into the role of cytonuclear interactions in crop domestication.

## MATERIALS AND METHODS

### Assembly and annotation of grapevine pan‐mitochondrial and pan‐plastid genomes

To investigate the evolution dynamics of mitochondrial and plastid genomes during *Vitis* domestication, we collected 33 samples from four groups (Table S1). Thirty‐four grapevine mitochondrial and plastid genomes were assembled using long‐read sequencing data. The average coverage of reads was downsampled to ~10 Gb. First, the long‐read sequencing data were compared with the reference mitochondrial genome *Vitis vinifera* (NC_012119.1) using minimap2 v2.26 (Li, 2021), and the mapped reads were subsequently extracted to assemble the mitochondrial genome with Flye v2.9.2‐b1786 (Kolmogorov et al., 2019). A similar strategy as the mitochondrial assembly was employed for assembling the plastid genomes. The pan‐genomes of the grapevine mitochondrial genome and plastid genome were constructed in minigraph‐0.13 (r397) with an incremental graph generation mode (https://github.com/lh3/minigraph). Reference genomes for plastid assembly included *Vitis amurensis* (NC_031383.1), *Vitis heyneana* (NC_039796.1), *Vitis piasezkii* (NC_039789.1), *Vitis retordii* (NC_039788.1), *Vitis riparia* (NC_039680.1), *Vitis rotundifolia* (NC_023790.1), *Vitis rupestris* (NC_036764.1) and *Vitis vinifera* (NC_007957.1). Mitochondrial and plastid genomes were annotated with the Geseq tool (Tillich et al., 2017), and the final annotations were manually checked to ensure the correct positions of the start and stop codons.

The forward repeats, reverse repeats and palindromic repeats within the 33 grapevine mitochondrial genomes were identified using REPuter (Kurtz et al., 2001) from BiBiServ2 (https://bibiserv.cebitec.uni-bielefeld.de) with parameters set to “Edit distance” of 3 and “Minimal Repeat Size” of 30 bp. NUCmer v3.1 (Kurtz et al., 2004) was used for the collinearity analysis of the 13 grape mitochondrial genomes, and the results were visualized using Mummerplot (Marçais et al., 2018). To visually observe gene loss in the mitochondrial genome, we use gene_content.R script for visualization.

### Population genetic analysis

We utilized published genome resequencing data for 100 grape accessions (Table S6). The “Vs” mitogenome served as the reference for single nucleotide polymorphism (SNP) and insertion/deletion (INDEL) calling. Raw data was mapped to the reference genome using Burrows–Wheeler Alignment v0.7.17‐r1188 (BWA) (Li and Durbin, 2009). SAMtools v1.13 (Li et al., 2009) was used to convert the resulting SAM files into BAM format. Variant identification was performed using GATK v4.2.3.0 (McKenna et al., 2010). The individual gVCF files from the 100 accessions were consolidated into a single VCF file using GLnexus v1.2.7 with the DeepVariantWGS config (Yun et al., 2021). Finally, SNPs and INDELs with depths (DP) greater than 2 and maximum missing rates less than 0.1 were extracted for further analysis.

The nucleotide diversity (*π*) and fixation index *F*_ST_ were calculated by VCFtools v0.1.16 (Danecek et al., 2011) with a 1,000‐bp sliding window and 500‐bp steps in grapes. To further identify the regions with signals of selective sweeps in cultivated grapes, a criterion was established: the top *F*_ST_ must be greater than 0.1, and the *π*_wild_/*π*_cul_ ratio must be greater than 1 based on common SNPs in the grape mitogenomes.

The genomic databases with SIFT predictions were generated using the tool make‐SIFT‐db‐all.pl, which took genomic sequence data in FASTA format and a gene annotation file in GTF format as input (Vaser et al., 2016). The required protein database was downloaded from UniProt (2021) (https://www.uniprot.org/). Subsequently, SNP data in VCF format, along with the genomic databases, were input into SIFT 4G to assess deleterious mutations.

### SV frequency assessment and origin of the grape mitogenome

To assess the SV frequency across 100 grape accessions, BEDTools v2.18 (Quinlan, 2014) was used to compute SV mapping coverage. The SV was segmented into three parts (SV1, SV2, and SV3), with their respective read depths (Idep) and whole‐genome depths (Wdep) calculated. To correct for sequencing depth variations, the Idep/Wdep ratio was used to determine SV presence or absence. SV1 and SV3 ratios were categorized into low (absent) and high (present) levels. Notably, SV2, homologous to plastid sequences, was excluded from further analysis. The frequencies of SV1 and SV3 in various grape populations were then compared using a chi‐square test to identify significant differences.

To determine the origin of the SV sequences, we performed a BLASTN search to detect the homologous sequence in 33 *Vitis* mitochondrial and plastid and nuclear genomes. The inferred putative origin of the intracellular transfer and nuclear‐shared sequences were identified by performing BLASTN searches of mitogenomes against nuclear DNA, with an e‐value cutoff lower than 1e‐10, identity greater than 90 and hit length more than 100 bp, and the ggplot2 package v3.4.3 (Villanueva and Chen, 2019) (https://cran.r-project.org/web/packages/ggplot2/index.html) was used to visualize the results.

### Identification of NUMTs and NUPTs for 13 grape species

First, Blastn v2.11.0+ (Camacho et al., 2009) was used to identify potential transfer events from organelles to the nuclear genome with the filter parameter “identity greater than 80% and alignment length greater than 100 bp.” Second, the average depths of the nuclear genome, mitochondrial genome and plastid genome were calculated, preliminary NUMT and NUPT fragments were identified based on their calculated depths. Specifically, the theoretical value of the depth of the NUMTs region = average depth of the nuclear genome + average depth of the mitochondrial genome, and similarly the theoretical value of the depth of the NUPTs region = average depth of the nuclear genome + average depth of the chloroplast genome. Finally, minimap2 v2.26 was used to map the 2 kb upstream and downstream sequences of NUMT and NUPT fragments to PacBio HiFi reads. The results were visualized in IGV v.2.12.3 to see if there were long reads linking NUMTs and NUPTs with their surrounding nuclear genomes, providing further evidence of NUMT and NUPT authenticity (Figures S24, S25).

### NUMTs and NUPTs dynamic insertion analysis

The distribution.R script was used to visualize the distribution of NUMTs and NUPTs on the nuclear genome. BEDtools helped to annotate the distribution patterns of NUMTs and NUPTs within different chromosomes and genomic regions (i.e., exon, intron et al.) based on the nuclear genome annotation file. The genome‐wide annotation pipeline and the identification of centromeres were adapted from previous research (Shi et al., 2023; Yue et al., 2023). Transposable element families were identified by RepeatModeler2 (Flynn et al., 2020) with a pan‐*Vitis* database of repeat families (Shi et al., 2023) and annotated all repetitive sequences of the combing reference genome using RepeatMasker v4.1.2 (Tarailo‐Graovac and Chen, 2009). The ggplot2 package v3.4.3 was used to visualize the results.

We then conducted a blastn search for two haplotypes of the same variety to identify both heterozygous and homozygous fragments of NUMTs and NUPTs. To detect the function of genes captured in the two haplotype regions of NUMTs and NUPTs, we downloaded the protein sequence library of Swiss‐Prot (https://ftp.ncbi.nlm.nih.gov/blast/db/FASTA/) for a local blast. After this, we extracted the NUMTs and NUPTs protein sequences for each haplotype of 13 grape varieties and blasted using diamond v.2.0.15 (parameter: –k 1 –e 0.00001, https://github.com/python-diamond/Diamond). We further extracted all genes of PN_T2T through diamond as background genes for GO and KEGG analysis. Then we uploaded the Swiss‐Prot ID to DAVID (https://david.ncifcrf.gov/tools.jsp) and completed GO enrichment and KEGG analysis. The results were further selected with the filter parameter *P*‐value threshold of less than 0.05. Finally, data visualization was completed by GO.R and KEGG.R scripts.

In addition, the One Step MCScanX tool within TBtools (Chen et al., 2023) was used to identify homologous gene pairs between wild and cultivated grapes. TBtools was also employed to calculate the Ka/Ks ratio (non‐synonymous to synonymous substitution ratio) for both heterozygous and homozygous NUMT genes between wild and cultivated grapes, and then a *t*‐test was used to test whether there were significant differences.

### The mapping of candidate genes associated with cytonuclear interactions in grapevine

We selected the samples used in the above artificial selection analysis to conduct GWAS analysis. There are two populations: cultivated (*n* = 50) and wild (*n* = 50). The pipeline for detecting nuclear genome variation was similar to that of the mitochondrial genome. Fastp v.0.23.4 (Chen, 2023) was used to filter low‐quality reads with default parameters, and BWA was used to compare the filtered data with the PN_T2T reference genome. The filtered BAM files were further processed by sorting with SAMtools v1.13 and marking the PCR duplicates with Picard v2.23.0 (http://broadinstitute.github.io/picard/), respectively. Subsequently, SNPs and InDels were identified using GATK v.4.2.6.0. This was followed by the consolidation of the genotyping from the gVCF files into a VCF file. High‐quality variants were obtained using PLINK with MAF ≥ 0.05 and missing < 0.2, resulting in 147,035 variants (137,077 SNPs, 9,958 InDels) for further analysis.

To eliminate the risk of false positives arising from the presence of multiple copies, we assessed the depth of coverage for each mitochondrial variant and compared it to the average depth across the whole mitochondrial genome. Variants with coverage depths exceeding 1.5 times the mean depth were excluded (Lian et al., 2024). Furthermore, we filtered out mitochondrial SNPs that were supported by fewer than four reads. Subsequently, we calculated the reference allele frequency by dividing the count of reads that support the reference allele by the total number of reads, and we term this value the “phenotype.” For the plastid, the phenotypic value is obtained in a similar way to the mitochondrial, but the unique plastid sequence is used as the reference, and the rest of the steps are the same as calculating the mitochondrial phenotypic value.

A mixed linear model in GEMMA v.0.98.3 (Zhou and Stephens, 2012) was employed for GWAS analysis. Variant positions and p‐wald test statistics (*P*‐value) were extracted to generate Manhattan and Q–Q plots using R scripts. A Python script was utilized to identify the associated genes within 10 kb windows of the significant variants, which were above the significance threshold of −Log_10_(0.05/Variant Numbers). GO enrichment analysis was employed using the web tool DAVID and the results were visualized with the GO.R script. The zoomed‐in plots of GWAS signals were visualized using LDBlockshow v.1.40 (Dong et al., 2021).

The salient sites associated with nuclear and mitochondria for each sample were screened out using VCFtools and the results were plotted with the pheatmap.R script. The chi‐squared test was used to test whether there were significant differences between wild grapes, cultivated grapes, and genotypes. The significantly associated loci and the non‐significantly associated loci were each screened out, and the proportion of NUMT events occurring at these sites was calculated.

## CONFLICTS OF INTEREST

The authors declare no conflicts of interest.

## AUTHOR CONTRIBUTIONS

Y.Z. and C.L. conceived and designed the study, and L.T. also provided assistance for the project. T.H. and C.L. performed experiments and data analysis. Y.X. and T.Z. collected the samples. Y.D., L.Z., C.C., Y.Z., X.S., and Y.L. assembled and annotated the haplotype‐resolved nuclear genomes. Y.P and H.X. provided the suggestions for genome selection and classification of gene structures, respectively. X.X., J.Y., Y.Z., X.S., J.L., M.D., X.F., S.Y., W.W., Z.C., S.Q., B.A., Z.J., and X.L. performed data analysis. T.H., C.L., and Y.X. wrote the manuscript. Y.Z. supervised the project. All authors provided critical feedback and revised the manuscript.

## Supporting information

Additional Supporting Information may be found online in the supporting information tab for this article: http://onlinelibrary.wiley.com/doi/10.1111/jipb.13968/suppinfo

**Figure S1.** Pan‐chloroplast structure from 33 *Vitis* accessions generated using Bandage
**Figure S2.** Pan‐chloroplast structure of *Vitis*
**Figure S3.** The gene content of 33 *Vitis* accessions
**Figure S4.** The pair‐wise synteny of mitochondrial genomes among Wine, Table, Syl (*Vitis vinifera* ssp. *sylvestris*) and Vwr (*Vitis* wild relatives) grape accessions
**Figure S5.** The depths of NUMTs and NUPTs of 13 species were identified
**Figure S6.** Phylogenetic tree topology in the grapes, the total number of NUMTs and NUPTs among genomes
**Figure S7.** The distribution of NUMTs in the nuclear chromosomes of table grapes
**Figure S8.** The distribution of NUMTs in the nuclear chromosomes of wine and wild grapes
**Figure S9.** The distribution of NUPTs in the nuclear chromosomes of table grapes
**Figure S10.** The distribution of NUPTs in the nuclear chromosomes of wine and wild grapes
**Figure S11.** Source sequence type of NUMTs for 13 grapes
**Figure S12.** Source sequence type of NUPTs for 13 grapes
**Figure S13.** Gene Ontology (GO) annotation of all genes captured in NUMTs
**Figure S14.** GO annotation of all genes captured in NUPTs
**Figure S15.** KEGG functional annotation of genes captured in NUMTs
**Figure S16.** KEGG functional annotation of genes captured in NUPTs
**Figure S17.** Manhattan plot of GWAS analysis for chloroplast SNPs in grapevine
**Figure S18.** The Q–Q plot of mitochondrial–nuclear interaction GWAS analysis (p_wald value)
**Figure S19.** The Q–Q plot of chloroplast–nuclear interaction GWAS analysis (p_wald value)
**Figure S20.** GO enrichment of mitochondria–nuclear interaction candidate genes
**Figure S21.** GO enrichment of chloroplast–nuclear interaction candidate genes
**Figure S22.** The grape mitochondrial genome size is related to copy number
**Figure S23.** Comparison of gene evolutionary rates (Ka/Ks) in three groups of wild and cultivated grapes
**Figure S24.** The IGV plot of NUMTs in grapes
**Figure S25.** The IGV plot of NUPTs in grapes

**Table S1.** The statistics of data used for organelle genome assembling and reads mapping
**Table S2.** Summary of 33 *Vitis* mitogenomes
**Table S3.** The TE annotation of 33 *Vitis* mitogenomes
**Table S4.** Summary of 33 *Vitis* chloroplast genomes
**Table S5.** The TE annotation of 33 *Vitis* chloroplast genomes
**Table S6.** Resequencing data of 100 grapes
**Table S7.** Mitochondrial genes in selective sweep regions
**Table S8.** Homologous sequence of SV (Vs: 665,448–695,774 bp) in 32 mitogenomes, chloroplast genomes and nuclear genomes
**Table S9.** The proportion of SV homologous sequences in 32 nuclear genomes
**Table S10.** The number of NUMTs/NUPTs between two haplotypes in 13 grapes
**Table S11.** The location of the four lost genes in NUPTs
**Table S12.** The list of genes predicted on NUMTs
**Table S13.** The list of genes predicted on NUPTs
**Table S14.** The annotation information of deleterious variation
**Table S15.** The mapping of NUMTs/NUPTs genes and deleterious genes in table grapes
**Table S16.** GO enrichment analysis of genes on NUMTs
**Table S17.** GO enrichment analysis of genes on NUPTs
**Table S18.** Predicted centromere regions in NUMTs
**Table S19.** Predicted centromere regions in NUPTs

## Data Availability

The PacBio data used for mitogenome and plastid genome assembly, along with the raw WGS data for grape accessions, were downloaded from NCBI. BioProject IDs are provided in Tables S1, S6, respectively. Mitochondrial and plastid genome assemblies and annotations of genome and variation maps were uploaded to Zenodo (https://zenodo.org/record/14489445). Custom scripts and workflows are available in the GitHub repository (https://github.com/zhouyflab/Nuclear_Cytoplasmic_Interactions/).

## References

[jipb13968-bib-0001] Aalto, E.A. , Koelewijn, H.P. , and Savolainen, O. (2013). Cytoplasmic male sterility contributes to hybrid incompatibility between subspecies of *Arabidopsis lyrata* . G3 (Bethesda) 3: 1727–1740. 23935000 10.1534/g3.113.007815 PMC3789797

[jipb13968-bib-0002] Adams, K.L. , Daley, D.O. , Qiu, Y.L. , Whelan, J. , and Palmer, J.D. (2000). Repeated, recent and diverse transfers of a mitochondrial gene to the nucleus in flowering plants. Nature 408: 354–357. 11099041 10.1038/35042567

[jipb13968-bib-0003] Adams, K.L. , Rosenblueth, M. , Qiu, Y.L. , and Palmer, J.D. (2001). Multiple losses and transfers to the nucleus of two mitochondrial succinate dehydrogenase genes during angiosperm evolution. Genetics 158: 1289–1300. 11454775 10.1093/genetics/158.3.1289 PMC1461739

[jipb13968-bib-0004] Baack, E. , Melo, M.C. , Rieseberg, L.H. , and Ortiz‐Barrientos, D. (2015). The origins of reproductive isolation in plants. New Phytol. 207: 968–984. 25944305 10.1111/nph.13424

[jipb13968-bib-0005] Barnard‐Kubow, K.B. , So, N. , and Galloway, L.F. (2016). Cytonuclear incompatibility contributes to the early stages of speciation. Evolution 70: 2752–2766. 27677969 10.1111/evo.13075

[jipb13968-bib-0006] Bogdanova, V.S. , and Galieva, E.R. (2009). Meiotic abnormalities as expression of nuclear‐cytoplasmic incompatibility in crosses of *Pisum sativum* subspecies. Genetika 45: 711–716. 19534431

[jipb13968-bib-0007] Burton, R.S. , and Barreto, F.S. (2012). A disproportionate role for mtDNA in Dobzhansky‐Muller incompatibilities? Mol. Ecol. 21: 4942–4957. 22994153 10.1111/mec.12006

[jipb13968-bib-0008] Camacho, C. , Coulouris, G. , Avagyan, V. , Ma, N. , Papadopoulos, J. , Bealer, K. , and Madden, T.L. (2009). BLAST+: Architecture and applications. BMC Bioinformatics 10: 421. 20003500 10.1186/1471-2105-10-421 PMC2803857

[jipb13968-bib-0009] Caruso, C.M. , Case, A.L. , and Bailey, M.F. (2012). The evolutionary ecology of cytonuclear interactions in angiosperms. Trends Plant Sci. 17: 638–643. 22784826 10.1016/j.tplants.2012.06.006

[jipb13968-bib-0010] Chase, C.D. (2007). Cytoplasmic male sterility: A window to the world of plant mitochondrial‐nuclear interactions. Trends Genet. 23: 81–90. 17188396 10.1016/j.tig.2006.12.004

[jipb13968-bib-0011] Chen, S. (2023). Ultrafast one‐pass FASTQ data preprocessing, quality control, and deduplication using fastp. Imeta 2: e107. 38868435 10.1002/imt2.107 PMC10989850

[jipb13968-bib-0012] Chen, C. , Wu, Y. , Li, J. , Wang, X. , Zeng, Z. , Xu, J. , Liu, Y. , Feng, J. , Chen, H. , He, Y. , et al. (2023). TBtools‐II: A “one for all, all for one” bioinformatics platform for biological big‐data mining. Mol. Plant 16: 1733–1742. 37740491 10.1016/j.molp.2023.09.010

[jipb13968-bib-0013] Cole, L.W. , Guo, W. , Mower, J.P. , and Palmer, J.D. (2018). High and variable rates of repeat‐mediated mitochondrial genome rearrangement in a genus of plants. Mol. Biol. Evol. 35: 2773–2785. 30202905 10.1093/molbev/msy176

[jipb13968-bib-0014] Consortium, T.R.C.S. (2003). In‐depth view of structure, activity, and evolution of rice chromosome 10. Science 300: 1566–1569. 12791992 10.1126/science.1083523

[jipb13968-bib-0015] Danecek, P. , Auton, A. , Abecasis, G. , Albers, C.A. , Banks, E. , DePristo, M.A. , Handsaker, R.E. , Lunter, G. , Marth, G.T. , Sherry, S.T. , et al. (2011). The variant call format and VCFtools. Bioinformatics 27: 2156–2158. 21653522 10.1093/bioinformatics/btr330 PMC3137218

[jipb13968-bib-0016] Dayama, G. , Zhou, W. , Prado‐Martinez, J. , Marques‐Bonet, T. , and Mills, R.E. (2020). Characterization of nuclear mitochondrial insertions in the whole genomes of primates. NAR Genom. Bioinform. 2: lqaa089. 33575633 10.1093/nargab/lqaa089 PMC7671390

[jipb13968-bib-0017] Dong, S.S. , He, W.M. , Ji, J.J. , Zhang, C. , Guo, Y. , and Yang, T.L. (2021). LDBlockShow: A fast and convenient tool for visualizing linkage disequilibrium and haplotype blocks based on variant call format files. Brief. Bioinform. 22: bbaa227. 33126247 10.1093/bib/bbaa227

[jipb13968-bib-0018] Dong, Y. , Duan, S. , Xia, Q. , Liang, Z. , Dong, X. , Margaryan, K. , Musayev, M. , Goryslavets, S. , Zdunić, G. , Bert, P.F. , et al. (2023). Dual domestications and origin of traits in grapevine evolution. Science 379: 892–901. 36862793 10.1126/science.add8655

[jipb13968-bib-0019] Flynn, J.M. , Hubley, R. , Goubert, C. , Rosen, J. , Clark, A.G. , Feschotte, C. , and Smit, A.F. (2020). RepeatModeler2 for automated genomic discovery of transposable element families. Proc. Natl. Acad. Sci. U. S. A. 117: 9451–9457. 32300014 10.1073/pnas.1921046117 PMC7196820

[jipb13968-bib-0020] Goremykin, V.V. , Salamini, F. , Velasco, R. , and Viola, R. (2009). Mitochondrial DNA of *Vitis vinifera* and the issue of rampant horizontal gene transfer. Mol. Biol. Evol. 26: 99–110. 18922764 10.1093/molbev/msn226

[jipb13968-bib-0021] Grassi, F. , and De Lorenzis, G. (2021). Back to the origins: Background and perspectives of grapevine domestication. Int. J. Mol. Sci. 22: 4518. 33926017 10.3390/ijms22094518 PMC8123694

[jipb13968-bib-0022] Gualberto, J.M. , and Newton, K.J. (2017). Plant mitochondrial genomes: Dynamics and mechanisms of mutation. Annu. Rev. Plant Biol. 68: 225–252. 28226235 10.1146/annurev-arplant-043015-112232

[jipb13968-bib-0023] Guo, L. , Wang, X. , Ayhan, D.H. , Rhaman, M.S. , Yan, M. , Jiang, J. , Wang, D. , Zheng, W. , Mei, J. , Ji, W. , et al. (2025). Super pangenome of Vitis empowers identification of downy mildew resistance genes for grapevine improvement. Nat. Genet. 57: 741–753. 40011682 10.1038/s41588-025-02111-7

[jipb13968-bib-0024] Hou, S. , Liu, D. , Huang, S. , Luo, D. , Liu, Z. , Xiang, Q. , Wang, P. , Mu, R. , Han, Z. , Chen, S. , et al. (2021). The Arabidopsis MIK2 receptor elicits immunity by sensing a conserved signature from phytocytokines and microbes. Nat. Commun. 12: 5494. 34535661 10.1038/s41467-021-25580-w PMC8448819

[jipb13968-bib-0025] Kan, S. , Liao, X. , Lan, L. , Kong, J. , Wang, J. , Nie, L. , Zou, J. , An, H. , and Wu, Z. (2024). Cytonuclear interactions and subgenome dominance shape the evolution of organelle‐targeted genes in the *Brassica Triangle* of U. Mol. Biol. Evol. 41: msae043. 38391484 10.1093/molbev/msae043 PMC10919925

[jipb13968-bib-0026] Keeling, P.J. (2010). The endosymbiotic origin, diversification and fate of plastids. Philos. Trans. R. Soc. Lond. B Biol. Sci. 365: 729–748. 20124341 10.1098/rstb.2009.0103 PMC2817223

[jipb13968-bib-0027] Kim, D.H. , Kang, J.G. , and Kim, B.D. (2007). Isolation and characterization of the cytoplasmic male sterility‐associated orf456 gene of chili pepper (*Capsicum annuum* L.). Plant Mol. Biol. 63: 519–532. 17238047 10.1007/s11103-006-9106-y

[jipb13968-bib-0028] Kleine, T. , Maier, U.G. , and Leister, D. (2009). DNA transfer from organelles to the nucleus: The idiosyncratic genetics of endosymbiosis. Annu. Rev. Plant Biol. 60: 115–138. 19014347 10.1146/annurev.arplant.043008.092119

[jipb13968-bib-0029] Kolmogorov, M. , Yuan, J. , Lin, Y. , and Pevzner, P.A. (2019). Assembly of long, error‐prone reads using repeat graphs. Nat. Biotechnol. 37: 540–546. 30936562 10.1038/s41587-019-0072-8

[jipb13968-bib-0030] Kurtz, S. , Choudhuri, J.V. , Ohlebusch, E. , Schleiermacher, C. , Stoye, J. , and Giegerich, R. (2001). REPuter: The manifold applications of repeat analysis on a genomic scale. Nucleic Acids Res. 29: 4633–4642. 11713313 10.1093/nar/29.22.4633 PMC92531

[jipb13968-bib-0031] Kurtz, S. , Phillippy, A. , Delcher, A.L. , Smoot, M. , Shumway, M. , Antonescu, C. , and Salzberg, S.L. (2004). Versatile and open software for comparing large genomes. Genome Biol. 5: R12. 14759262 10.1186/gb-2004-5-2-r12 PMC395750

[jipb13968-bib-0032] Leister, D. (2005). Origin, evolution and genetic effects of nuclear insertions of organelle DNA. Trends Genet. 21: 655–663. 16216380 10.1016/j.tig.2005.09.004

[jipb13968-bib-0033] Leon, P. , Arroyo, A. , and Mackenzie, S. (1998). Nuclear control of plastid and mitochondrial development in higher plants. Annu. Rev. Plant Physiol. Plant Mol. Biol. 49: 453–480. 15012242 10.1146/annurev.arplant.49.1.453

[jipb13968-bib-0034] Levin, D.A. (2003). The cytoplasmic factor in plant speciation. Syst. Bot. 28: 5–11.

[jipb13968-bib-0035] Lexer, C. , Welch, M.E. , Raymond, O. , and Rieseberg, L.H. (2003). The origin of ecological divergence in Helianthus paradoxus (Asteraceae): Selection on transgressive characters in a novel hybrid habitat. Evolution 57: 1989–2000. 14575321 10.1111/j.0014-3820.2003.tb00379.x

[jipb13968-bib-0036] Li, H. (2021). New strategies to improve minimap2 alignment accuracy. Bioinformatics 37: 4572–4574. 34623391 10.1093/bioinformatics/btab705 PMC8652018

[jipb13968-bib-0037] Li, H. , and Durbin, R. (2009). Fast and accurate short read alignment with Burrows–Wheeler transform. Bioinformatics 25: 1754–1760. 19451168 10.1093/bioinformatics/btp324 PMC2705234

[jipb13968-bib-0038] Li, H. , Handsaker, B. , Wysoker, A. , Fennell, T. , Ruan, J. , Homer, N. , Marth, G. , Abecasis, G. , and Durbin, R. (2009). The sequence alignment/map format and SAMtools. Bioinformatics 25: 2078–2079. 19505943 10.1093/bioinformatics/btp352 PMC2723002

[jipb13968-bib-0039] Lian, Q. , Li, S. , Kan, S. , Liao, X. , Huang, S. , Sloan, D.B. , and Wu, Z. (2024). Association analysis provides insights into plant mitonuclear interactions. Mol. Biol. Evol. 41: msae028. 38324417 10.1093/molbev/msae028 PMC10875325

[jipb13968-bib-0040] Lin, X. , Kaul, S. , Rounsley, S. , Shea, T.P. , Benito, M.I. , Town, C.D. , Fujii, C.Y. , Mason, T. , Bowman, C.L. , Barnstead, M. , et al. (1999). Sequence and analysis of chromosome 2 of the plant *Arabidopsis thaliana* . Nature 402: 761–768. 10617197 10.1038/45471

[jipb13968-bib-0041] Liu, Z. , Wang, N. , Su, Y. , Long, Q. , Peng, Y. , Shangguan, L. , Zhang, F. , Cao, S. , Wang, X. , Ge, M. , et al. (2024). Grapevine pangenome facilitates trait genetics and genomic breeding. Nat. Genet. 56: 2804–2814. 39496880 10.1038/s41588-024-01967-5 PMC11631756

[jipb13968-bib-0042] Magdy, M. , Ou, L. , Yu, H. , Chen, R. , Zhou, Y. , Hassan, H. , Feng, B. , Taitano, N. , van der Knaap, E. , Zou, X. , et al. (2019). Pan‐plastome approach empowers the assessment of genetic variation in cultivated *Capsicum* species. Hortic. Res. 6: 108. 31645963 10.1038/s41438-019-0191-x PMC6804749

[jipb13968-bib-0043] Marçais, G. , Delcher, A.L. , Phillippy, A.M. , Coston, R. , Salzberg, S.L. , and Zimin, A. (2018). MUMmer4: A fast and versatile genome alignment system. PLoS Comput. Biol. 14: e1005944. 29373581 10.1371/journal.pcbi.1005944 PMC5802927

[jipb13968-bib-0044] McKenna, A. , Hanna, M. , Banks, E. , Sivachenko, A. , Cibulskis, K. , Kernytsky, A. , Garimella, K. , Altshuler, D. , Gabriel, S. , Daly, M. , et al. (2010). The genome analysis toolkit: A MapReduce framework for analyzing next‐generation DNA sequencing data. Genome Res. 20: 1297–1303. 20644199 10.1101/gr.107524.110 PMC2928508

[jipb13968-bib-0045] Michalovova, M. , Vyskot, B. , and Kejnovsky, E. (2013). Analysis of plastid and mitochondrial DNA insertions in the nucleus (NUPTs and NUMTs) of six plant species: Size, relative age and chromosomal localization. Heredity (Edinb) 111: 314–320. 23715017 10.1038/hdy.2013.51 PMC3807264

[jipb13968-bib-0046] Noutsos, C. , Richly, E. , and Leister, D. (2005). Generation and evolutionary fate of insertions of organelle DNA in the nuclear genomes of flowering plants. Genome Res. 15: 616–628. 15867426 10.1101/gr.3788705 PMC1088290

[jipb13968-bib-0047] Peng, Y. , Wang, Y. , Liu, Y. , Fang, X. , Cheng, L. , Long, Q. , Su, D. , Zhang, T. , Shi, X. , Xu, X. , et al. (2025). The genomic and epigenomic landscapes of hemizygous genes across crops with contrasting reproductive systems. Proc. Natl. Acad. Sci. U. S. A. 122: e2422487122. 39918952 10.1073/pnas.2422487122 PMC11831139

[jipb13968-bib-0048] Perea‐Resa, C. , Rodríguez‐Milla, M.A. , Iniesto, E. , Rubio, V. , and Salinas, J. (2017). Prefoldins negatively regulate cold acclimation in *Arabidopsis thaliana* by promoting nuclear proteasome‐mediated HY5 degradation. Mol. Plant 10: 791–804. 28412546 10.1016/j.molp.2017.03.012

[jipb13968-bib-0049] Purugganan, M.D. , and Fuller, D.Q. (2009). The nature of selection during plant domestication. Nature 457: 843–848. 19212403 10.1038/nature07895

[jipb13968-bib-0050] Quinlan, A.R. (2014). BEDTools: The Swiss‐Army tool for genome feature analysis. Curr. Protoc. Bioinformatics 47: 11.12.1–34. 10.1002/0471250953.bi1112s47 PMC4213956 25199790

[jipb13968-bib-0051] Richly, E. , and Leister, D. (2004). NUPTs in sequenced eukaryotes and their genomic organization in relation to NUMTs. Mol. Biol. Evol. 21: 1972–1980. 15254258 10.1093/molbev/msh210

[jipb13968-bib-0052] Roger, A.J. , Muñoz‐Gómez, S.A. , and Kamikawa, R. (2017). The origin and diversification of mitochondria. Curr. Biol. 27: R1177–R1192. 29112874 10.1016/j.cub.2017.09.015

[jipb13968-bib-0053] Roux, F. , Mary‐Huard, T. , Barillot, E. , Wenes, E. , Botran, L. , Durand, S. , Villoutreix, R. , Martin‐Magniette, M.L. , Camilleri, C. , and Budar, F. (2016). Cytonuclear interactions affect adaptive traits of the annual plant *Arabidopsis thaliana* in the field. Proc. Natl. Acad. Sci. U. S. A. 113: 3687–3692. 26979961 10.1073/pnas.1520687113 PMC4822599

[jipb13968-bib-0054] Sambatti, J.B. , Ortiz‐Barrientos, D. , Baack, E.J. , and Rieseberg, L.H. (2008). Ecological selection maintains cytonuclear incompatibilities in hybridizing sunflowers. Ecol. Lett. 11: 1082–1091. 18643842 10.1111/j.1461-0248.2008.01224.x PMC2737365

[jipb13968-bib-0055] Schmitz‐Linneweber, C. , Kushnir, S. , Babiychuk, E. , Poltnigg, P. , Herrmann, R.G. , and Maier, R.M. (2005). Pigment deficiency in nightshade/tobacco cybrids is caused by the failure to edit the plastid ATPase alpha‐subunit mRNA. Plant Cell 17: 1815–1828. 15894714 10.1105/tpc.105.032474 PMC1143079

[jipb13968-bib-0056] Sharbrough, J. , Conover, J.L. , Tate, J.A. , Wendel, J.F. , and Sloan, D.B. (2017). Cytonuclear responses to genome doubling. Am. J. Bot. 104: 1277–1280. 29885242 10.3732/ajb.1700293

[jipb13968-bib-0057] Shi, X. , Cao, S. , Wang, X. , Huang, S. , Wang, Y. , Liu, Z. , Liu, W. , Leng, X. , Peng, Y. , Wang, N. , et al. (2023). The complete reference genome for grapevine (*Vitis vinifera* L.) genetics and breeding. Hortic. Res. 10: uhad061. 37213686 10.1093/hr/uhad061 PMC10199708

[jipb13968-bib-0058] Sloan, D.B. , Havird, J.C. , and Sharbrough, J. (2017). The on‐again, off‐again relationship between mitochondrial genomes and species boundaries. Mol. Ecol. 26: 2212–2236. 27997046 10.1111/mec.13959 PMC6534505

[jipb13968-bib-0059] Sloan, D.B. , Warren, J.M. , Williams, A.M. , Wu, Z. , Abdel‐Ghany, S.E. , Chicco, A.J. , and Havird, J.C. (2018). Cytonuclear integration and co‐evolution. Nat. Rev. Genet. 19: 635–648. 30018367 10.1038/s41576-018-0035-9 PMC6469396

[jipb13968-bib-0060] Stahl, E. , Fernandez Martin, A. , Glauser, G. , Guillou, M.C. , Aubourg, S. , Renou, J.P. , and Reymond, P. (2022). The MIK2/SCOOP signaling system contributes to *Arabidopsis* resistance against herbivory by modulating jasmonate and indole glucosinolate biosynthesis. Front. Plant Sci. 13: 852808. 35401621 10.3389/fpls.2022.852808 PMC8984487

[jipb13968-bib-0061] Stupar, R.M. , Lilly, J.W. , Town, C.D. , Cheng, Z. , Kaul, S. , Buell, C.R. , and Jiang, J. (2001). Complex mtDNA constitutes an approximate 620‐kb insertion on *Arabidopsis thaliana* chromosome 2: Implication of potential sequencing errors caused by large‐unit repeats. Proc. Natl. Acad. Sci. U. S. A. 98: 5099–5103. 11309509 10.1073/pnas.091110398 PMC33170

[jipb13968-bib-0062] Sun, M. , Zhang, M. , Chen, X. , Liu, Y. , Liu, B. , Li, J. , Wang, R. , Zhao, K. , and Wu, J. (2022). Rearrangement and domestication as drivers of Rosaceae mitogenome plasticity. BMC Biol. 20: 181. 35986276 10.1186/s12915-022-01383-3 PMC9392253

[jipb13968-bib-0063] Tarailo‐Graovac, M. , and Chen, N. (2009). Using RepeatMasker to identify repetitive elements in genomic sequences. Curr. Protoc. Bioinformatics Chapter 4: Unit 4.10. 10.1002/0471250953.bi0410s25 19274634

[jipb13968-bib-0064] Tillich, M. , Lehwark, P. , Pellizzer, T. , Ulbricht‐Jones, E.S. , Fischer, A. , Bock, R. , and Greiner, S. (2017). GeSeq—versatile and accurate annotation of organelle genomes. Nucleic Acids Res. 45: W6–W11. 28486635 10.1093/nar/gkx391 PMC5570176

[jipb13968-bib-0065] Timmis, J.N. , Ayliffe, M.A. , Huang, C.Y. , and Martin, W. (2004). Endosymbiotic gene transfer: Organelle genomes forge eukaryotic chromosomes. Nat. Rev. Genet. 5: 123–135. 14735123 10.1038/nrg1271

[jipb13968-bib-0066] Toriyama, K. (2021). Molecular basis of cytoplasmic male sterility and fertility restoration in rice. Plant Biotechnol (Tokyo) 38: 285–295. 34782814 10.5511/plantbiotechnology.21.0607a PMC8562580

[jipb13968-bib-0067] UniProt Consortium (2021). UniProt: The universal protein knowledgebase in 2021. Nucleic Acids Res. 49: D480–D489. 33237286 10.1093/nar/gkaa1100 PMC7778908

[jipb13968-bib-0068] Ureshino, K. , Abe, T. , and Akabane, M. (2010). Relationship between nuclear genome construction and the plastome–genome incompatibility of progenies from intra‐ and inter‐ploid cross of evergreen azaleas × Rhododendron japonicum f. flavum. J. Jpn. Soc. Hortic. Sci. 79: 91–96.

[jipb13968-bib-0069] Vaser, R. , Adusumalli, S. , Leng, S.N. , Sikic, M. , and Ng, P.C. (2016). SIFT missense predictions for genomes. Nat. Protoc. 11: 1–9. 26633127 10.1038/nprot.2015.123

[jipb13968-bib-0070] Villanueva, R.A.M. , Chen, and Z.J. (2019). Ggplot2: Elegant Graphics for Data Analysis. Taylor & Francis, UK.

[jipb13968-bib-0071] Wang, N. , Li, C. , Kuang, L. , Wu, X. , Xie, K. , Zhu, A. , Xu, Q. , Larkin, R.M. , Zhou, Y. , Deng, X. , et al. (2022). Pan‐mitogenomics reveals the genetic basis of cytonuclear conflicts in *citrus* hybridization, domestication, and diversification. Proc. Natl. Acad. Sci. U. S. A. 119: e2206076119. 36260744 10.1073/pnas.2206076119 PMC9618123

[jipb13968-bib-0072] Wei, W. , Schon, K.R. , Elgar, G. , Orioli, A. , Tanguy, M. , Giess, A. , Tischkowitz, M. , Caulfield, M.J. , and Chinnery, P.F. (2022). Nuclear‐embedded mitochondrial DNA sequences in 66,083 human genomes. Nature 611: 105–114. 36198798 10.1038/s41586-022-05288-7 PMC9630118

[jipb13968-bib-0073] Woodson, J.D. , and Chory, J. (2008). Coordination of gene expression between organellar and nuclear genomes. Nat. Rev. Genet. 9: 383–395. 18368053 10.1038/nrg2348 PMC4854206

[jipb13968-bib-0074] Xiao, H. , Liu, Z. , Wang, N. , Long, Q. , Cao, S. , Huang, G. , Liu, W. , Peng, Y. , Riaz, S. , Walker, A.M. , et al. (2023). Adaptive and maladaptive introgression in grapevine domestication. Proc. Natl. Acad. Sci. U. S. A. 120: e2222041120. 37276420 10.1073/pnas.2222041120 PMC10268302

[jipb13968-bib-0075] Xiao, H. , Wang, Y. , Liu, W. , Shi, X. , Huang, S. , Cao, S. , Long, Q. , Wang, X. , Liu, Z. , Xu, X. , et al. (2025). Impacts of reproductive systems on grapevine genome and breeding. Nat. Commun. 16: 2031. 40032836 10.1038/s41467-025-56817-7 PMC11876636

[jipb13968-bib-0076] Yang, H. , Xue, Y. , Li, B. , Lin, Y. , Li, H. , Guo, Z. , Li, W. , Fu, Z. , Ding, D. , and Tang, J. (2022). The chimeric gene *atp6c* confers cytoplasmic male sterility in maize by impairing the assembly of the mitochondrial ATP synthase complex. Mol. Plant 15: 872–886. 35272047 10.1016/j.molp.2022.03.002

[jipb13968-bib-0077] Yue, J. , Chen, Q. , Wang, Y. , Zhang, L. , Ye, C. , Wang, X. , Cao, S. , Lin, Y. , Huang, W. , Xian, H. , et al. (2023). Telomere‐to‐telomere and gap‐free reference genome assembly of the kiwifruit *Actinidia chinensis* . Hortic. Res. 10: uhac264. 36778189 10.1093/hr/uhac264 PMC9909506

[jipb13968-bib-0078] Yun, T. , Li, H. , Chang, P.C. , Lin, M.F. , Carroll, A. , and McLean, C.Y. (2021). Accurate, scalable cohort variant calls using DeepVariant and GLnexus. Bioinformatics 36: 5582–5589. 33399819 10.1093/bioinformatics/btaa1081 PMC8023681

[jipb13968-bib-0079] Zhang, S. , Wang, J. , He, W. , Kan, S. , Liao, X. , Jordan, D.R. , Mace, E.S. , Tao, Y. , Cruickshank, A.W. , Klein, R. , et al. (2023). Variation in mitogenome structural conformation in wild and cultivated lineages of *sorghum* corresponds with domestication history and plastome evolution. BMC Plant Biol. 23: 91. 36782130 10.1186/s12870-023-04104-2 PMC9926791

[jipb13968-bib-0080] Zhang, Z. , Zhao, J. , Li, J. , Yao, J. , Wang, B. , Ma, Y. , Li, N. , Wang, H. , Wang, T. , Liu, B. , et al. (2024). Evolutionary trajectory of organelle‐derived nuclear DNAs in the *Triticum/Aegilops* complex species. Plant Physiol. 194: 918–935. 37847157 10.1093/plphys/kiad552 PMC10828211

[jipb13968-bib-0081] Zhou, X. , and Stephens, M. (2012). Genome‐wide efficient mixed‐model analysis for association studies. Nat. Genet. 44: 821–824. 22706312 10.1038/ng.2310 PMC3386377

[jipb13968-bib-0082] Zhou, Y. , Massonnet, M. , Sanjak, J.S. , Cantu, D. , and Gaut, B.S. (2017). Evolutionary genomics of grape (*Vitis vinifera* ssp. *vinifera*) domestication. Proc. Natl. Acad. Sci. U. S. A. 114: 11715–11720. 29042518 10.1073/pnas.1709257114 PMC5676911

[jipb13968-bib-0083] Zhou, Y. , Muyle, A. , and Gaut, B.S. (2019). Evolutionary genomics and the domestication of grapes. In The Grape Genome. Cantu, D. , Walker, M.A. , eds, (Cham: Springer), pp. 39–55.

[jipb13968-bib-0084] Zu, X. , Luo, L. , Wang, Z. , Gong, J. , Yang, C. , Wang, Y. , Xu, C. , Qiao, X. , Deng, X. , Song, X. , et al. (2023). A mitochondrial pentatricopeptide repeat protein enhances cold tolerance by modulating mitochondrial superoxide in rice. Nat. Commun. 14: 6789. 37880207 10.1038/s41467-023-42269-4 PMC10600133

[jipb13968-bib-0085] Zupok, A. , Kozul, D. , Schöttler, M.A. , Niehörster, J. , Garbsch, F. , Liere, K. , Fischer, A. , Zoschke, R. , Malinova, I. , Bock, R. , et al. (2021). A photosynthesis operon in the chloroplast genome drives speciation in evening primroses. Plant Cell 33: 2583–2601. 34048579 10.1093/plcell/koab155 PMC8408503

