# Supplementary material for: The cytonuclear interactions during grapevine domestication
Source: J Integr Plant Biol. 2025 Jul 29;67(10):2686–703. doi: 10.1111/jipb.13968 (PMC12498075; doi:10.1111/jipb.13968)
Supplement: Supplementary file 1 — Figure S1. Pan‐chloroplast structure from 33 Vitis accessions generated using Bandage Figure S2. Pan‐chloroplast structure of Vitis Figure S3. The gene content of 33 Vitis accessions Figure S4. The pair‐wise synteny of mitochondrial genomes among Wine, Table, Syl (Vitis vinifera ssp. sylvestris) and Vwr (Vitis wild relatives) grape accessions Figure S5. The depths of NUMTs and NUPTs of 13 species were identified Figure S6. Phylogenetic tree topology in the grapes, the total number of NUMTs and NUPTs among genomes Figure S7. The distribution of NUMTs in the nuclear chromosomes of table grapes Figure S8. The distribution of NUMTs in the nuclear chromosomes of wine and wild grapes Figure S9. The distribution of NUPTs in the nuclear chromosomes of table grapes Figure S10. The distribution of NUPTs in the nuclear chromosomes of wine and wild grapes Figure S11. Source sequence type of NUMTs for 13 grapes Figure S12. Source sequence type of NUPTs for 13 grapes Figure S13. Gene Ontology (GO) annotation of all genes captured in NUMTs Figure S14. GO annotation of all genes captured in NUPTs Figure S15. KEGG functional annotation of genes captured in NUMTs Figure S16. KEGG functional annotation of genes captured in NUPTs Figure S17. Manhattan plot of GWAS analysis for chloroplast SNPs in grapevine Figure S18. The Q–Q plot of mitochondrial–nuclear interaction GWAS analysis (p_wald value) Figure S19. The Q–Q plot of chloroplast–nuclear interaction GWAS analysis (p_wald value) Figure S20. GO enrichment of mitochondria–nuclear interaction candidate genes Figure S21. GO enrichment of chloroplast–nuclear interaction candidate genes Figure S22. The grape mitochondrial genome size is related to copy number Figure S23. Comparison of gene evolutionary rates (Ka/Ks) in three groups of wild and cultivated grapes Figure S24. The IGV plot of NUMTs in grapes Figure S25. The IGV plot of NUPTs in grapes [file JIPB-67-2686-s001.docx]

Supplemental figures:


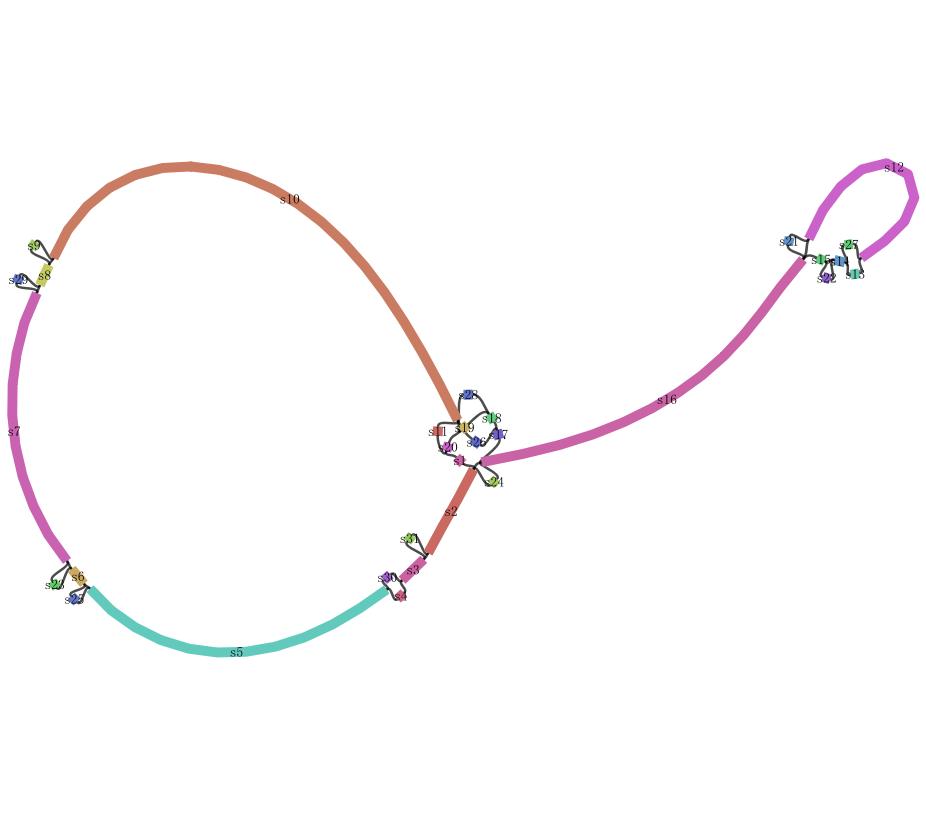


**Figure S1. Pan-chloroplast structure from 33 *Vitis* accessions generated using Bandage.**


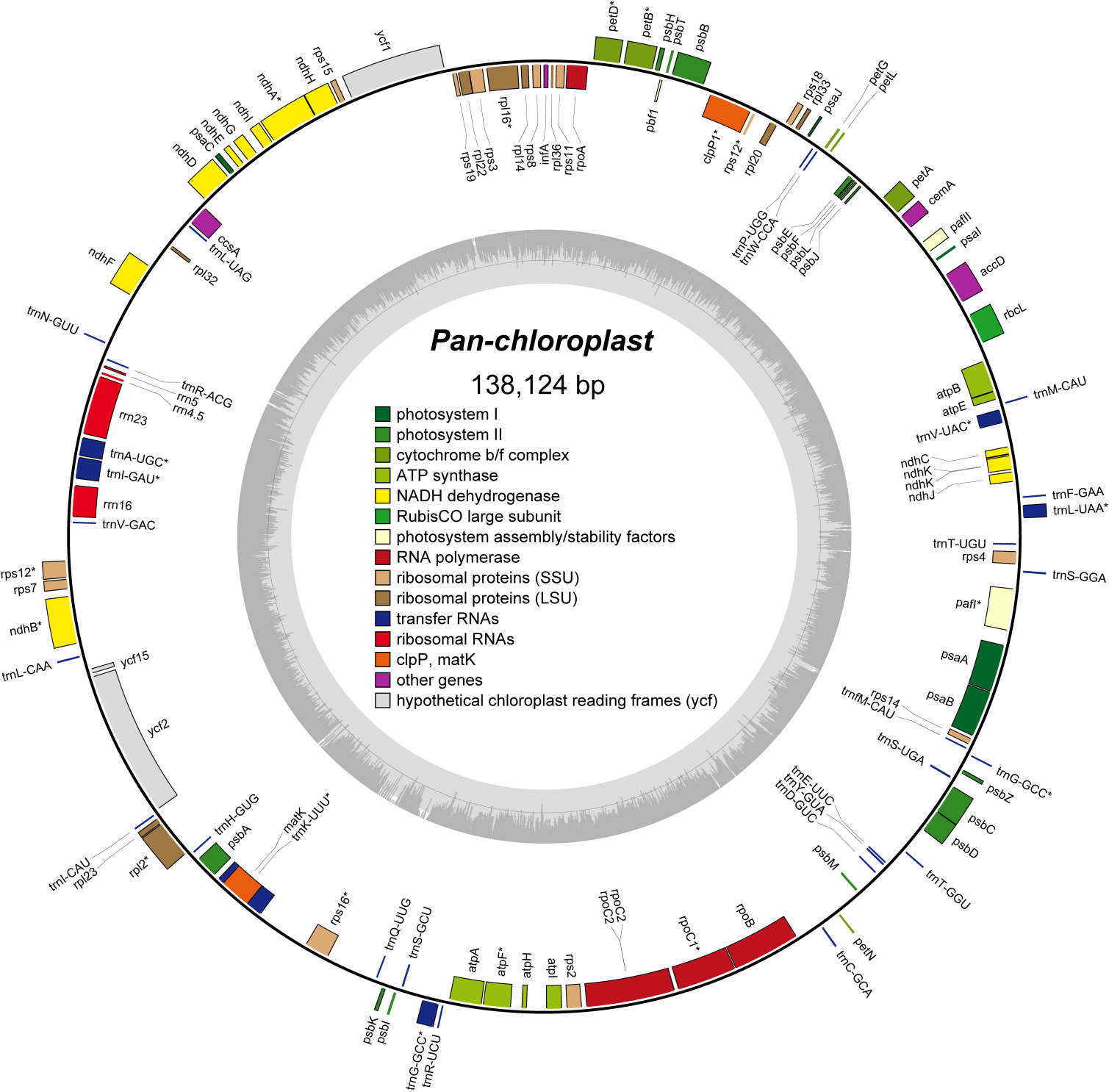


**Figure S2. Pan-chloroplast structure of *Vitis*.** Genes mapped outside the outer circle are transcribed counterclockwise, and those inside are transcribed clockwise. Genes are color coded by functional group.


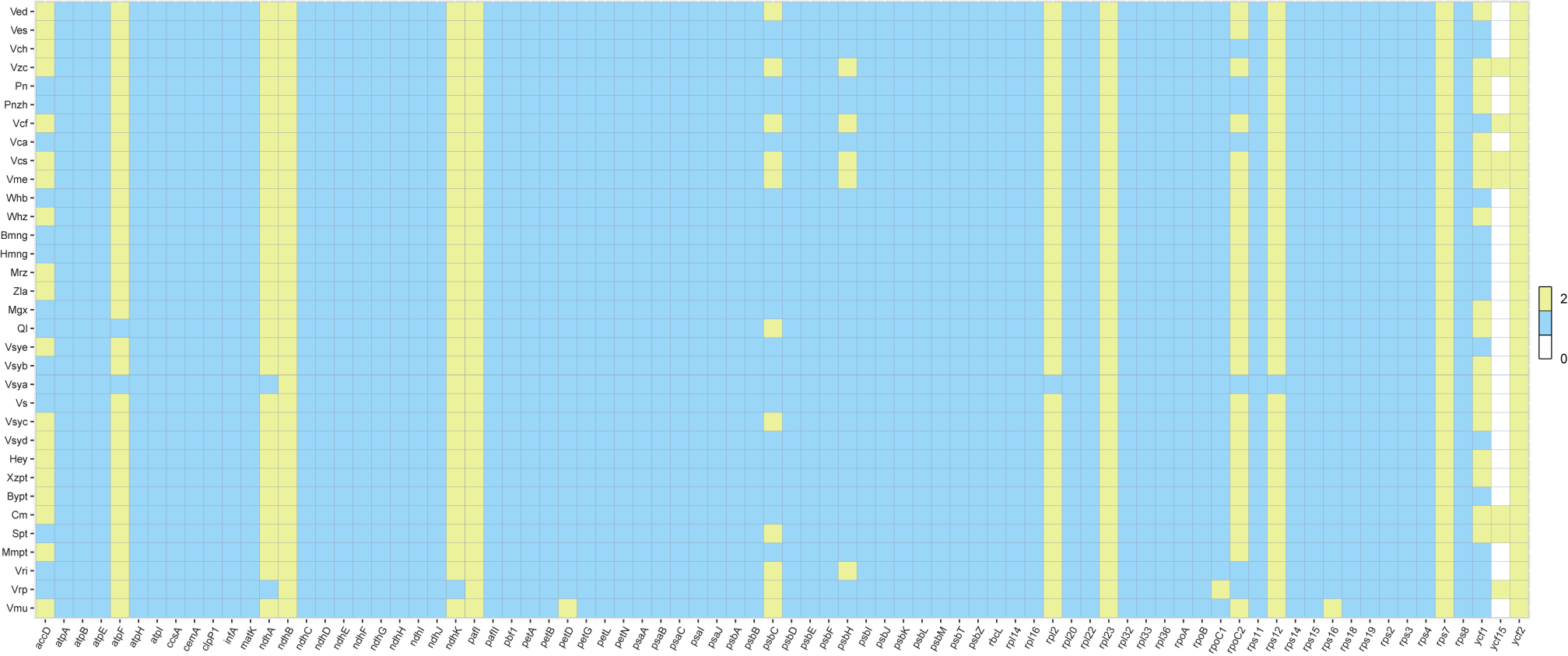
**Figure S3. The gene content of 33 *Vitis* accessions.** Different colors represent the number of genes present in each chloroplast genome.

**
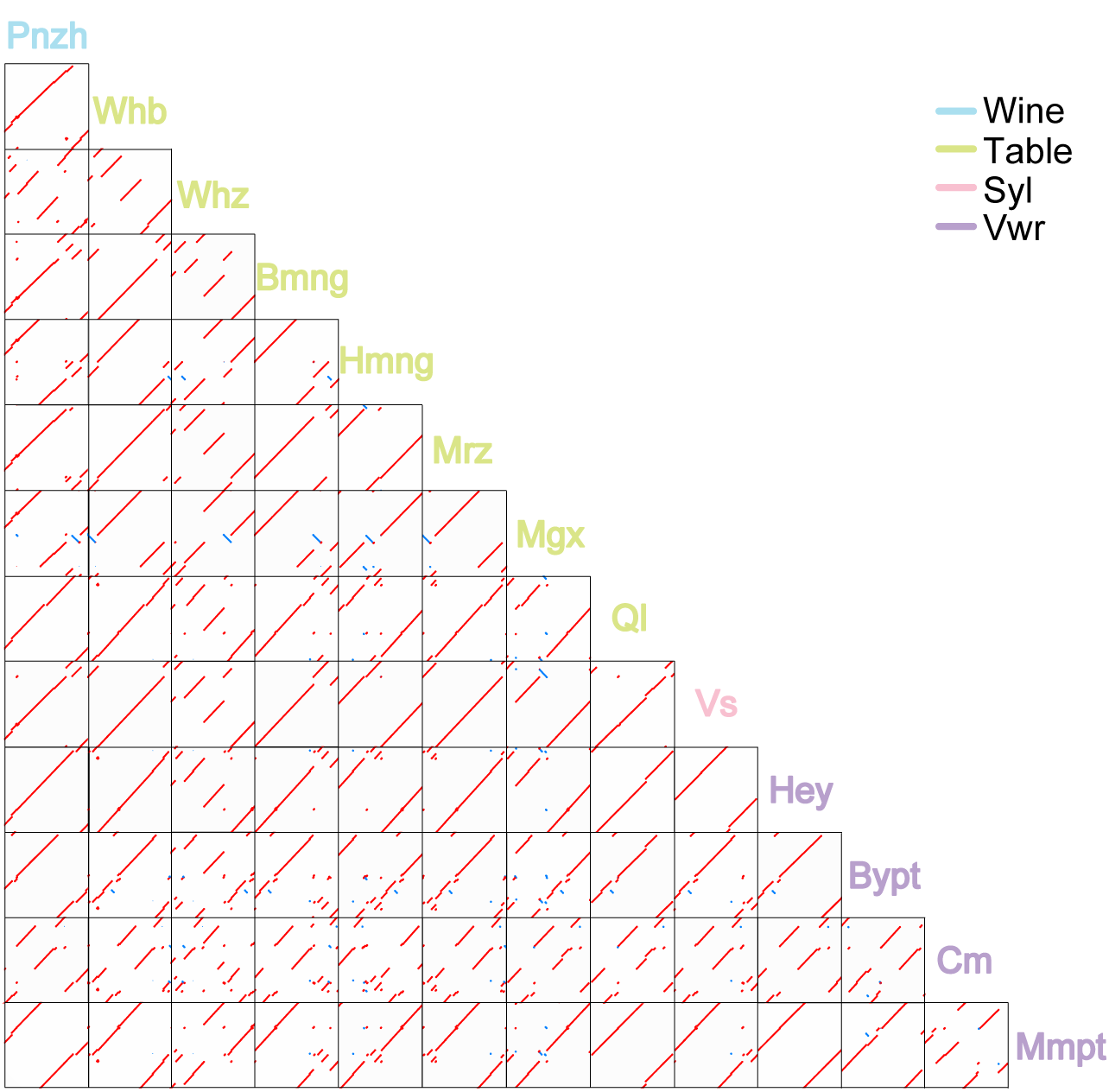
**

**Figure S4. The pair-wise synteny of mitochondrial genomes among Wine, Table, Syl (*Vitis vinifera* ssp. *sylvestris*) and Vwr (*Vitis* wild relatives) grape accessions.** The grapes in blue are wine grapes, the yellow-green ones are table grapes, the pink ones are Syl, and the purple ones are Vwr grapes. Red represents direct, and blue represents inverted.


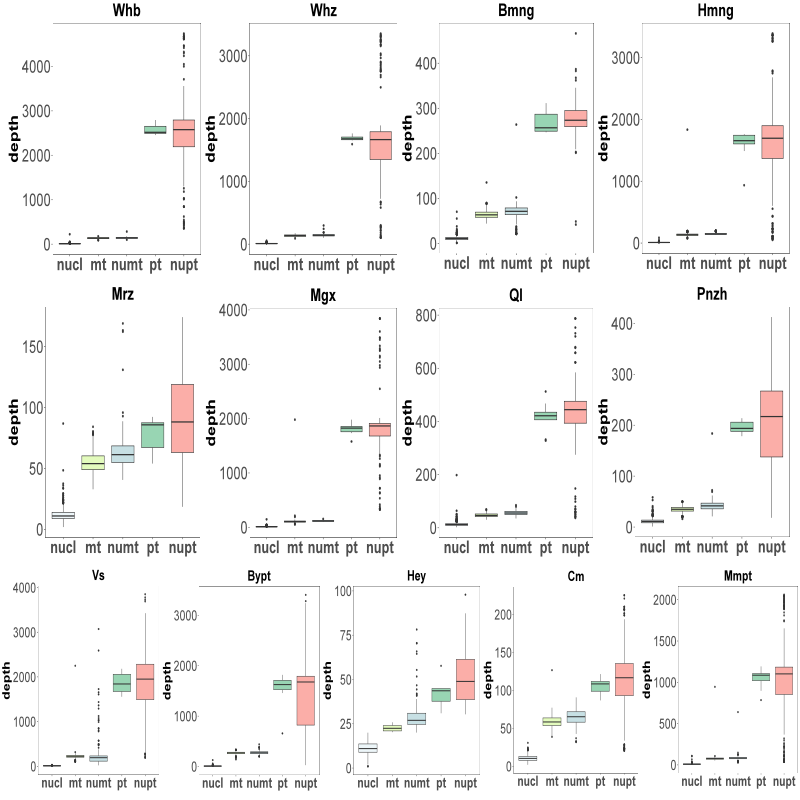


**Figure S5. The depths of NUMTs and NUPTs of 13 species were identified.** The horizontal coordinates represent the nuclear, mitochondrial, NUMT, chloroplast, and NUPT segments, respectively, and the vertical coordinates indicate the depth of these segments.


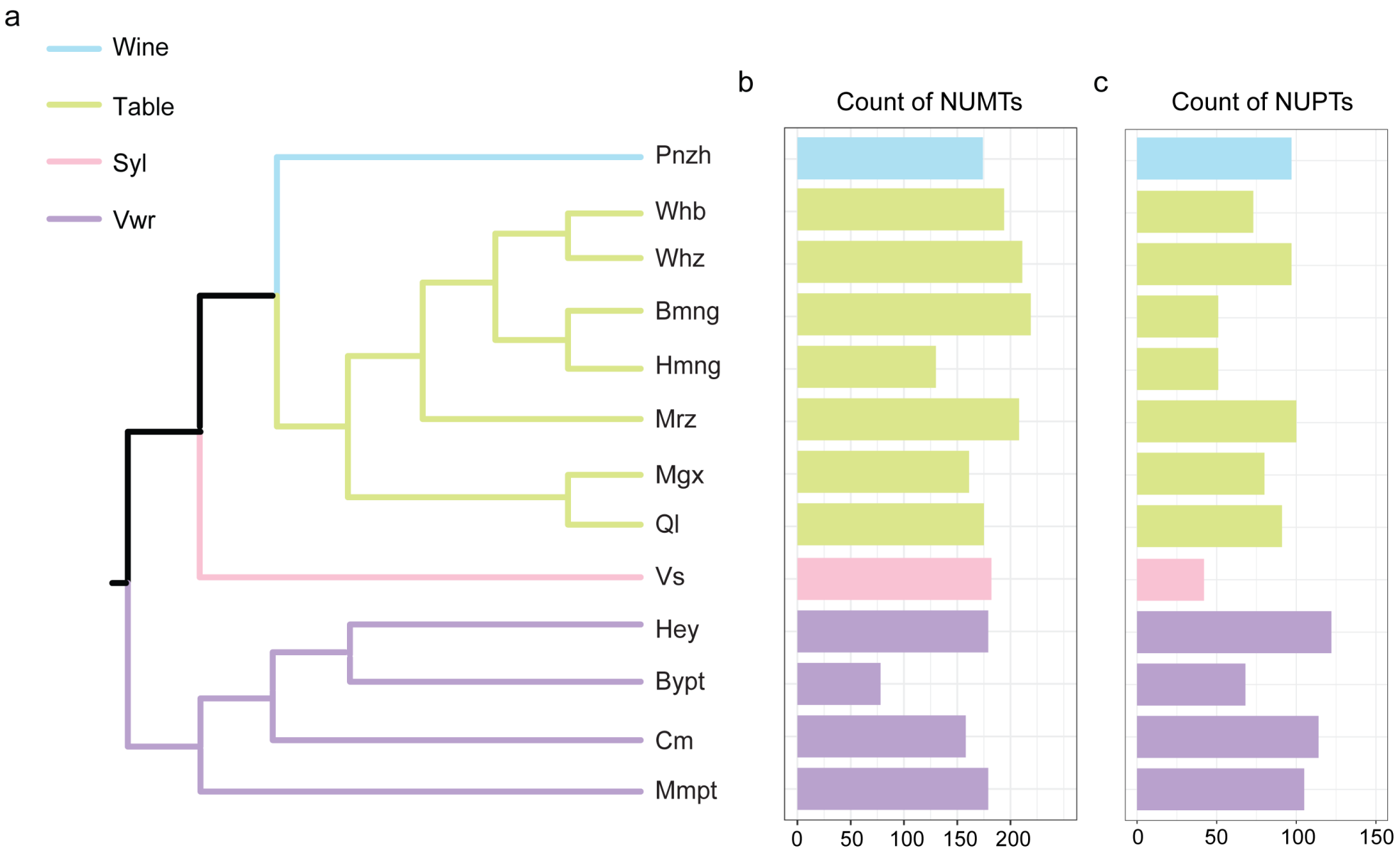


**Figure S6. Phylogenetic tree topology in the grapes (A), the total number of NUMTs (B) and NUPTs (C) among genomes.**


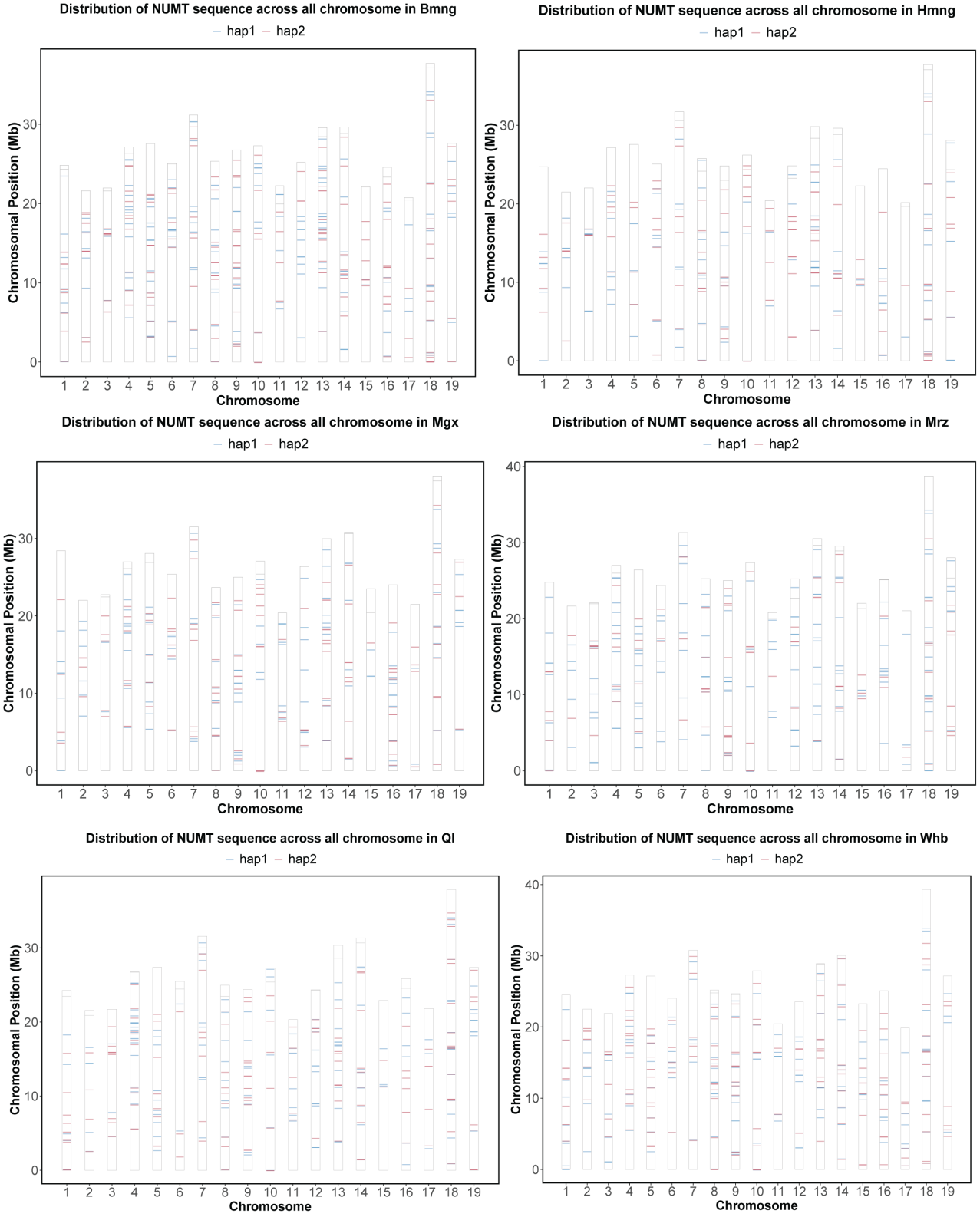


**Figure S7. The distribution of NUMTs in the nuclear chromosomes of table grapes.** The x-axis represents the 19 chromosomes of the grape, while the y-axis represents the location of the NUMT fragments on the chromosome, where hap1 is represented by the blue line and hap2 by the purple line.


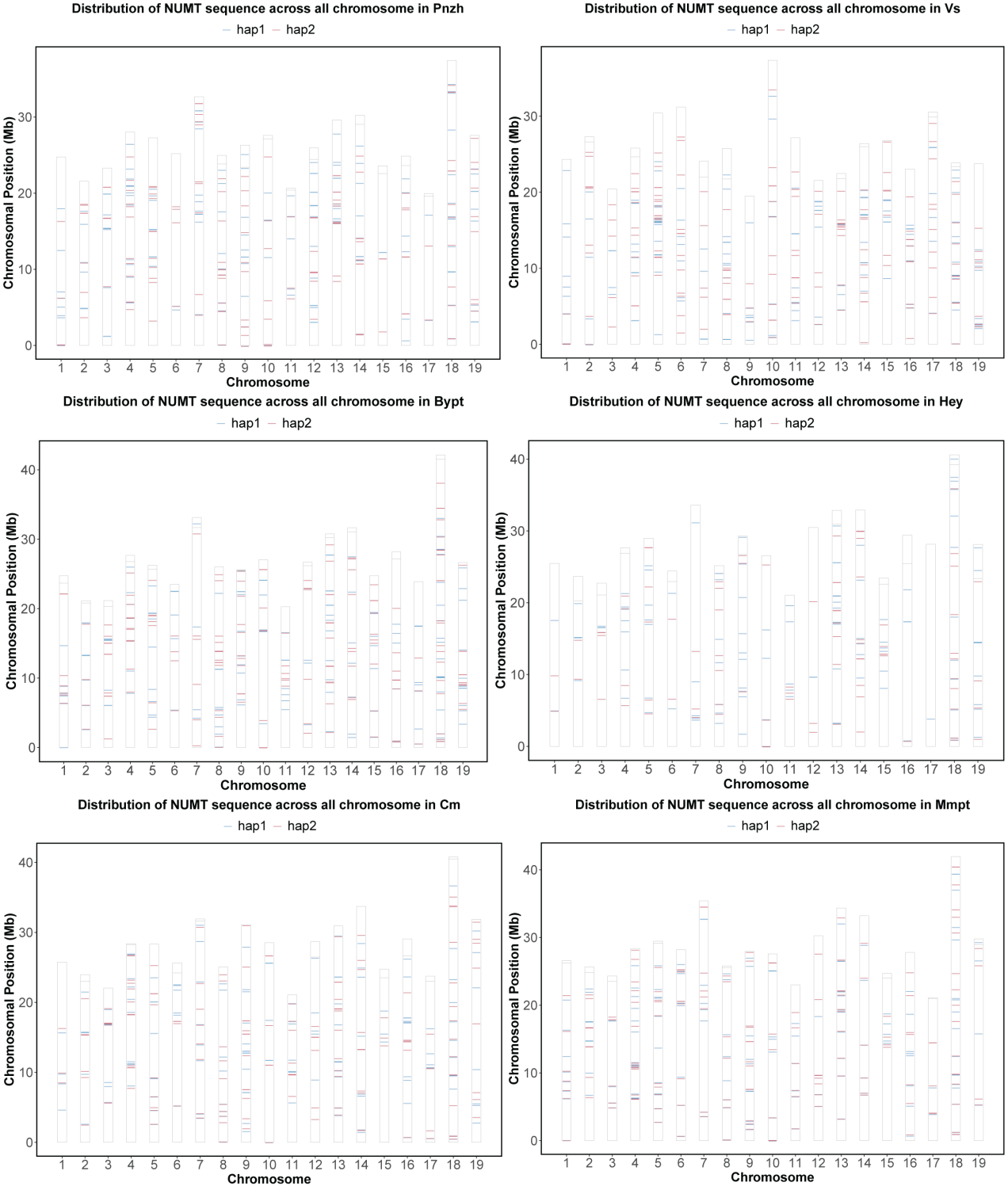


**Figure S8. The distribution of NUMTs in the nuclear chromosomes of wine and wild grapes.** The x-axis represents the 19 chromosomes of the grape, while the y-axis represents the location of the NUMT fragments on the chromosome, where hap1 is represented by the blue line and hap2 by the purple line.


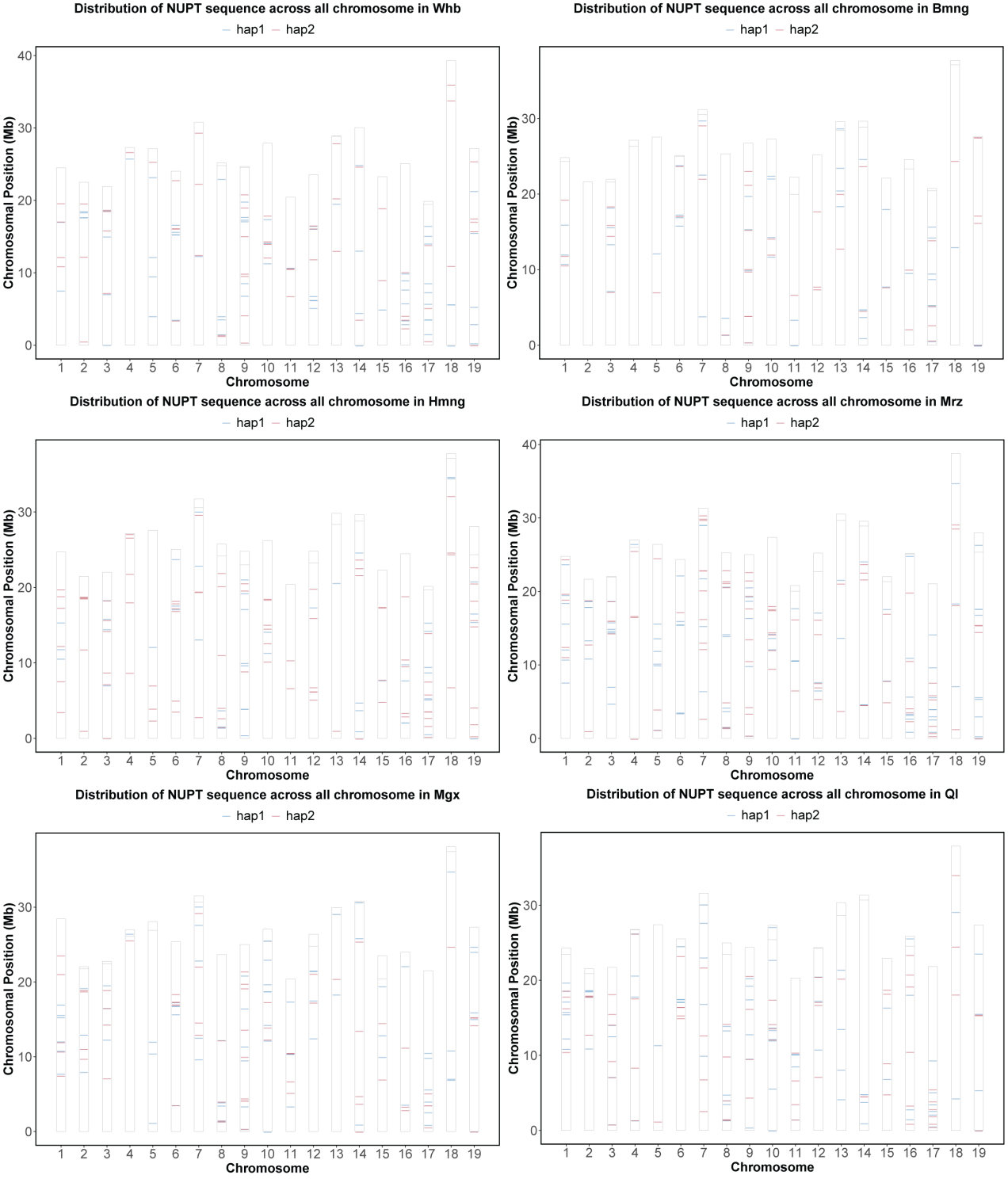


**Figure S9. The distribution of NUPTs in the nuclear chromosomes of table grapes.** The x-axis represents the 19 chromosomes of the grape, while the y-axis represents the location of the NUPT fragments on the chromosome, where hap1 is represented by the blue line and hap2 by the purple line.


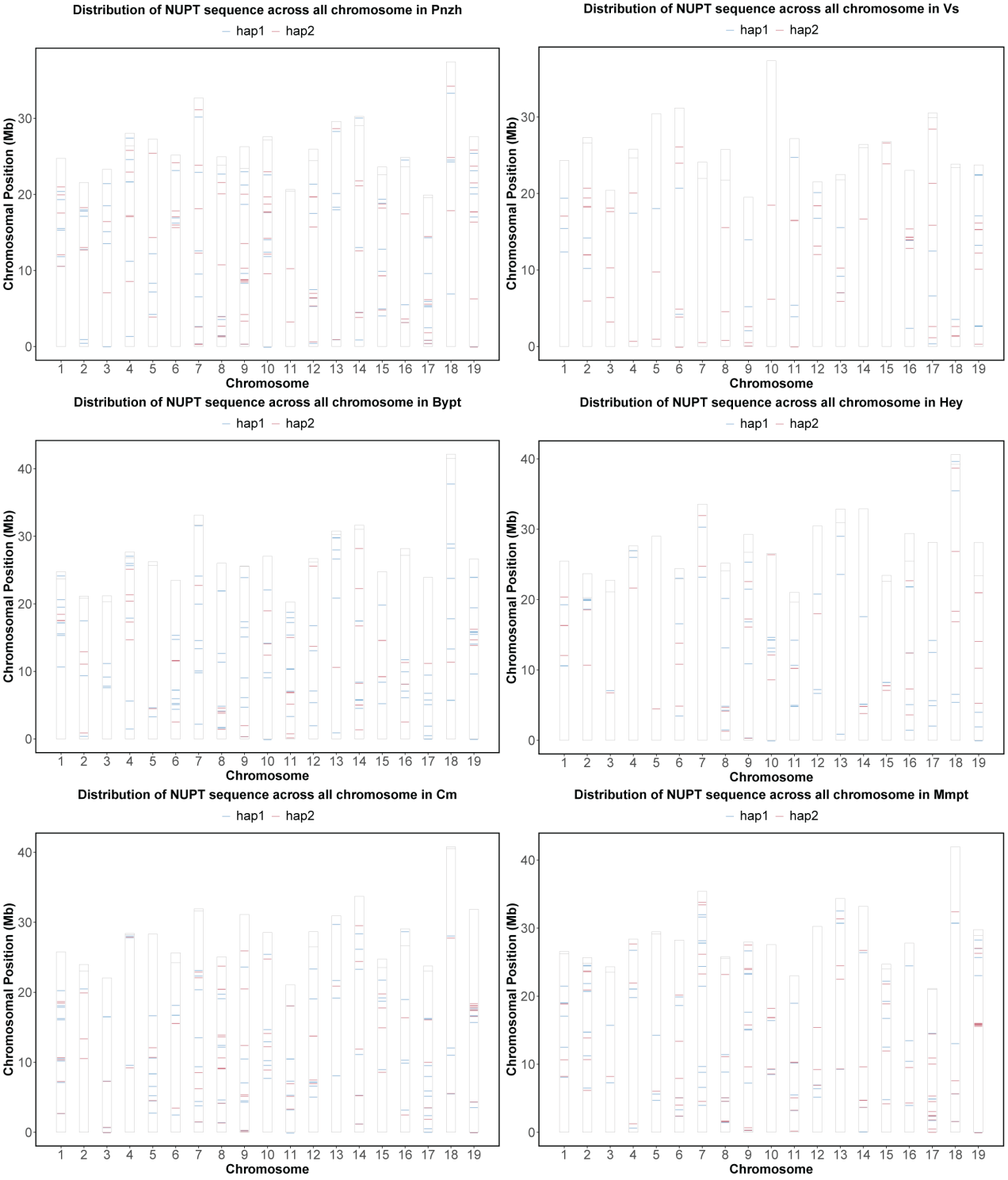


**Figure S10. The distribution of NUPTs in the nuclear chromosomes of wine and wild grapes.** The x-axis represents the 19 chromosomes of the grape, while the y-axis represents the location of the NUPT fragments on the chromosome, where hap1 is represented by the blue line and hap2 by the purple line.


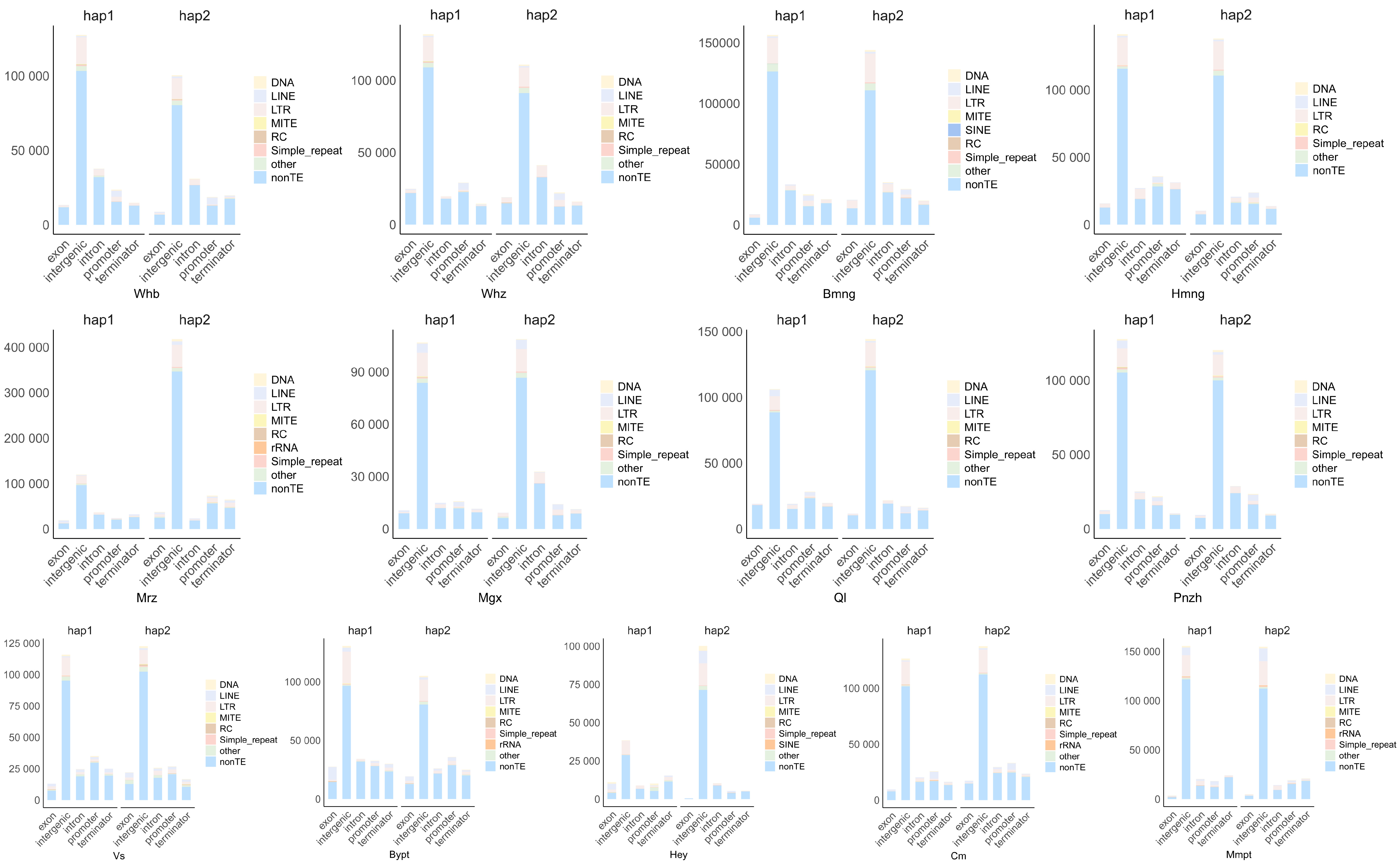
**Figure S11. Source sequence type of NUMTs for 13 grapes.** To the left, we observe haplotype 1, while on the right, haplotype 2 is presented. The x-axis illustrates various segments of the gene structure, and the y-axis denotes the sequence lengths of diverse types of transposable elements within these different gene structural components.


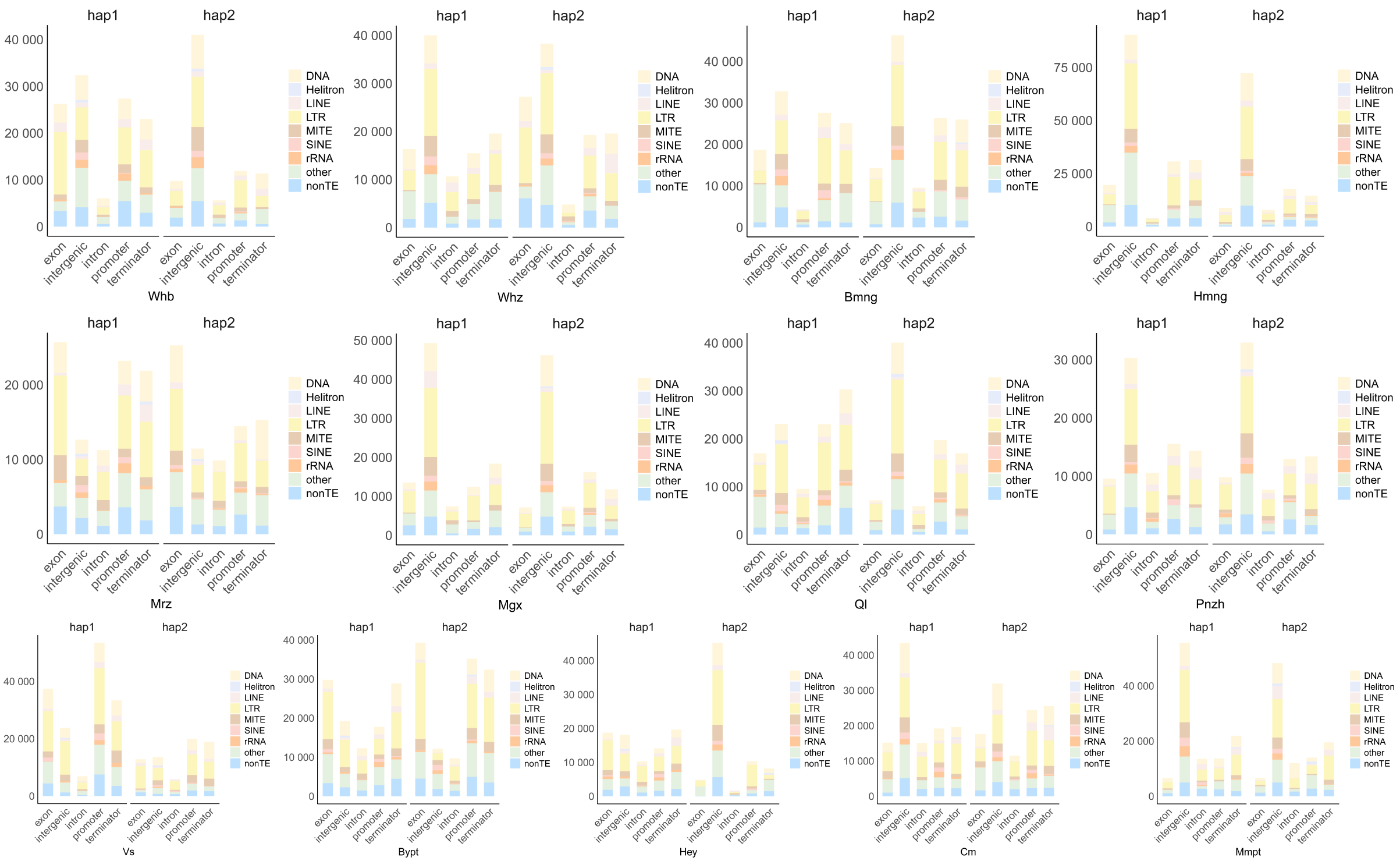
**Figure S12. Source sequence type of NUPTs for 13 grapes.** To the left, we observe haplotype 1, while on the right, haplotype 2 is presented. The x-axis illustrates various segments of the gene structure, and the y-axis denotes the sequence lengths of diverse types of transposable elements within these different gene structural components.


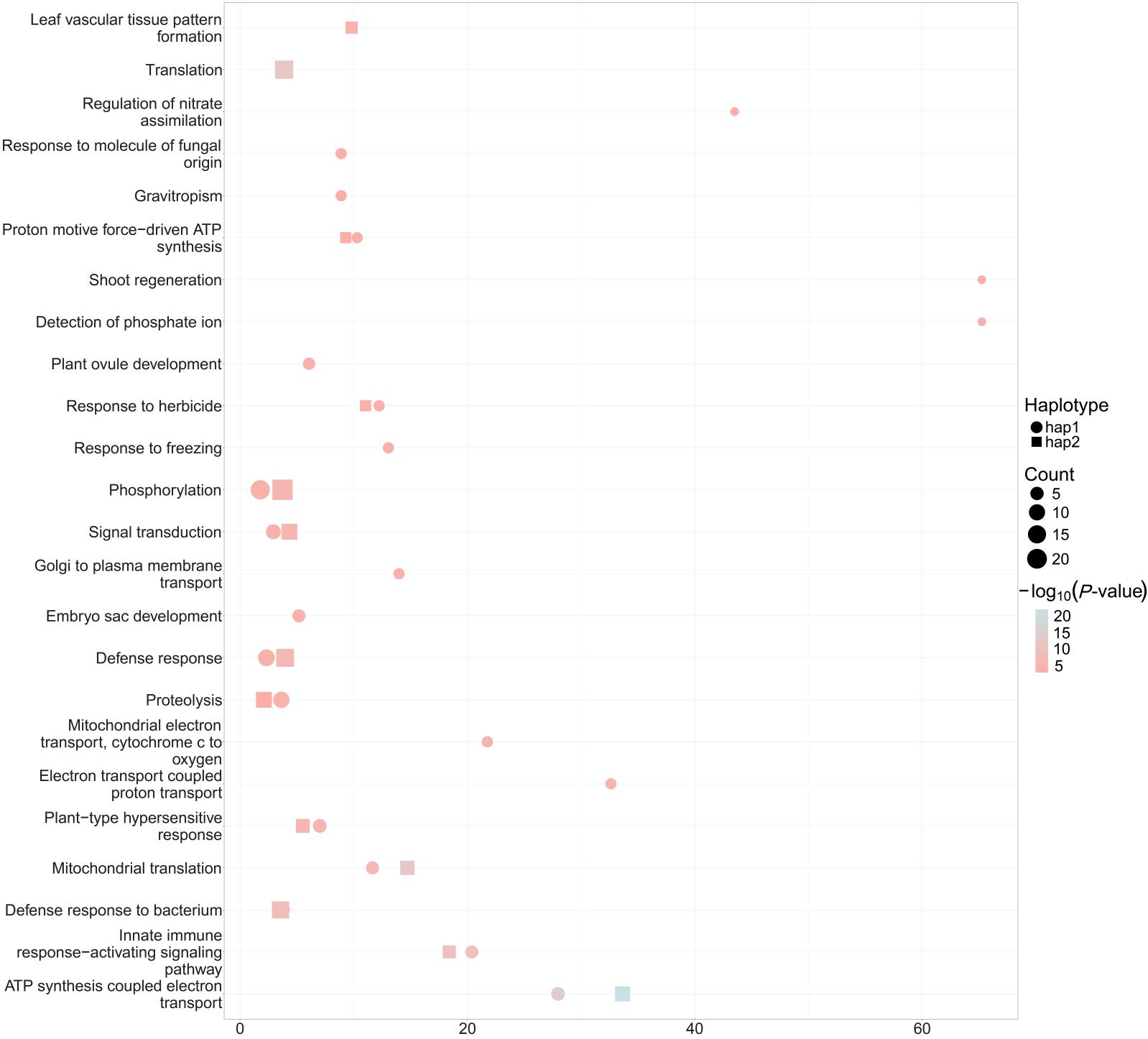


**Figure S13. GO annotation of all genes captured in NUMTs.** In the diagram, the circles represent hap1, while the squares represent hap2. The size of the circles indicates the number of genes, with larger circles indicating a greater number of genes.


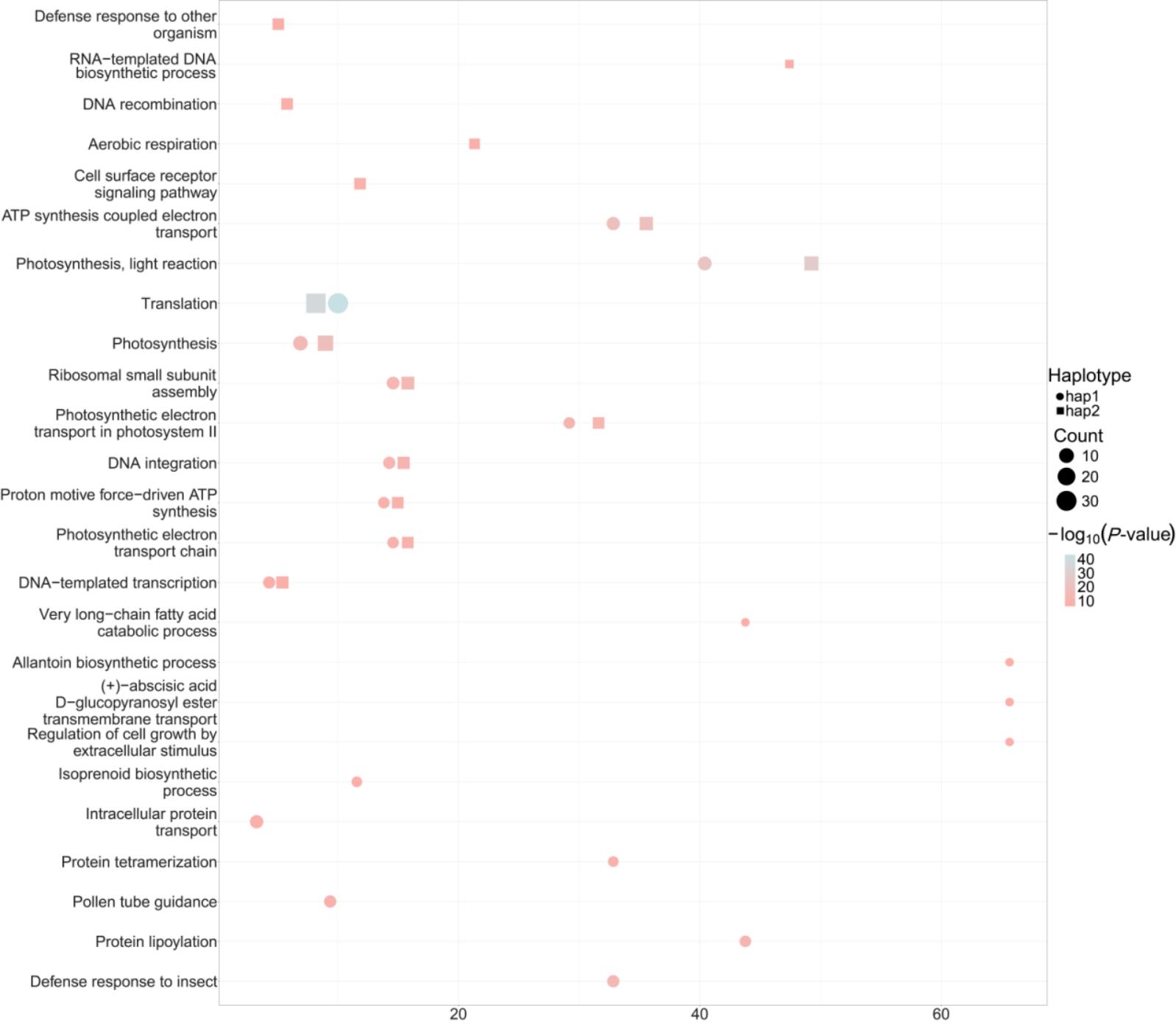


**Figure S14. GO annotation of all genes captured in NUPTs.** In the diagram, the circles represent hap1, while the squares represent hap2. The size of the circles indicates the number of genes, with larger circles indicating a greater number of genes.





**Figure S15. KEGG functional annotation of genes captured in NUMTs.** The metabolic pathways highlighted in red are those that are specific to hap2.


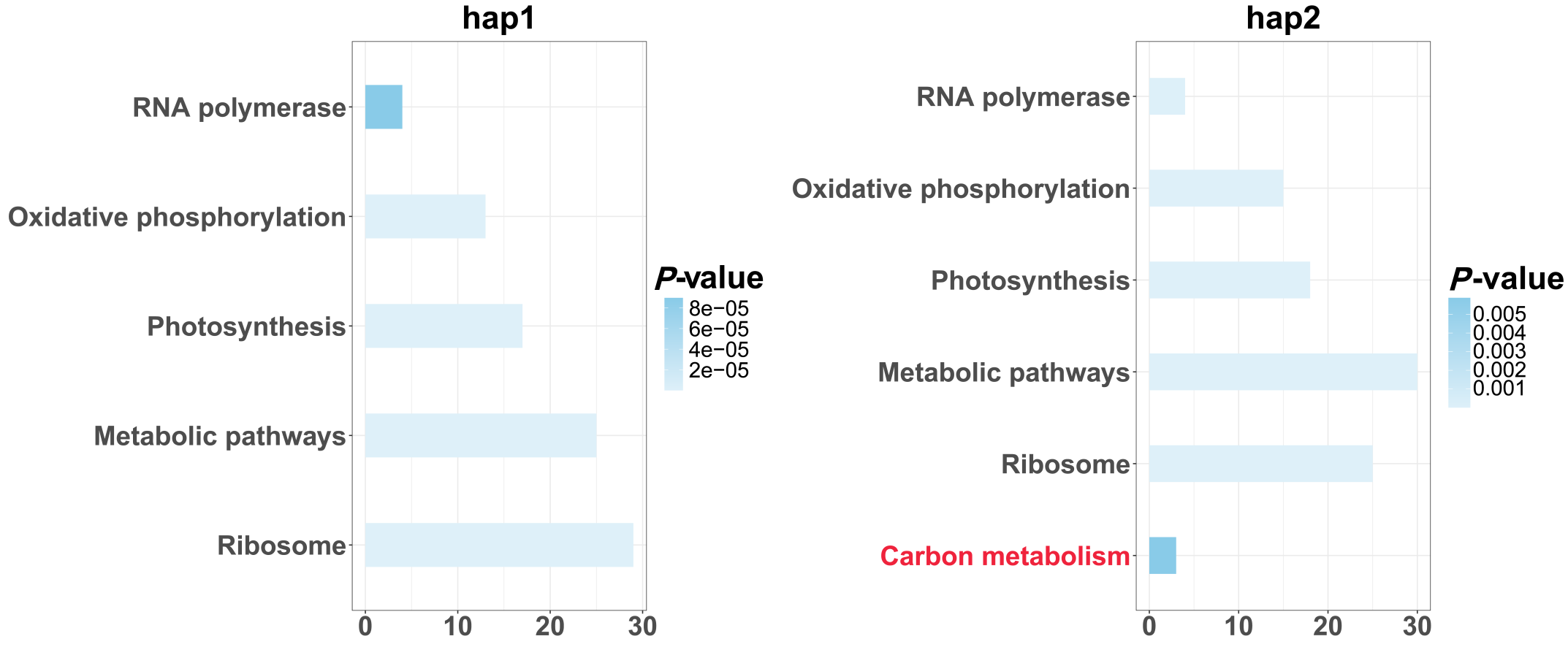


**Figure S16. KEGG functional annotation of genes captured in NUPTs.** The pathway highlighted in red is specific for hap2.


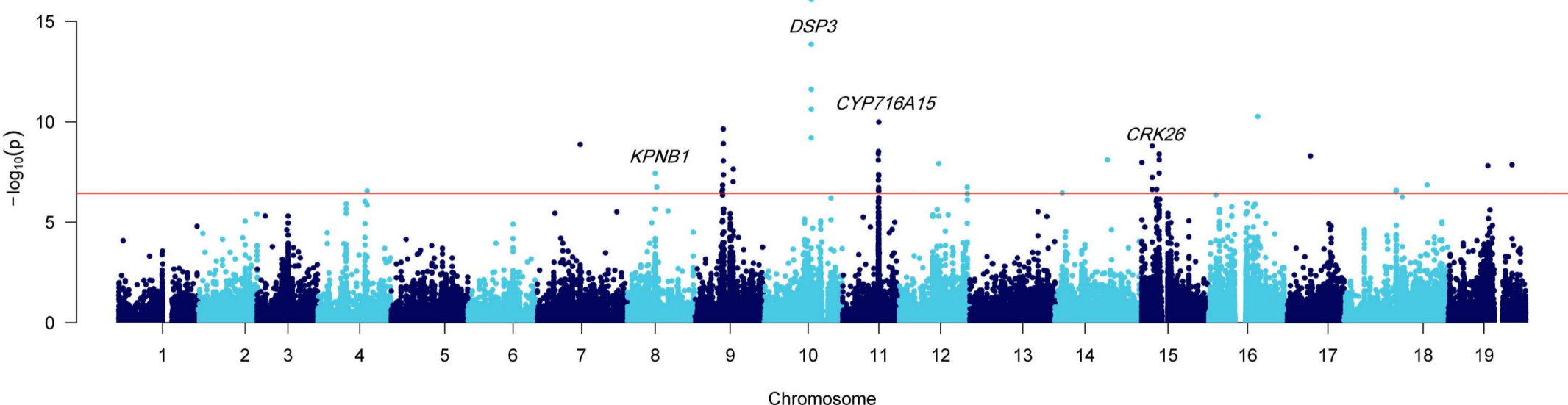


**Figure S17. Manhattan plot of GWAS analysis for chloroplast SNPs in grapevine.** The dashed horizontal line represents the significance threshold.


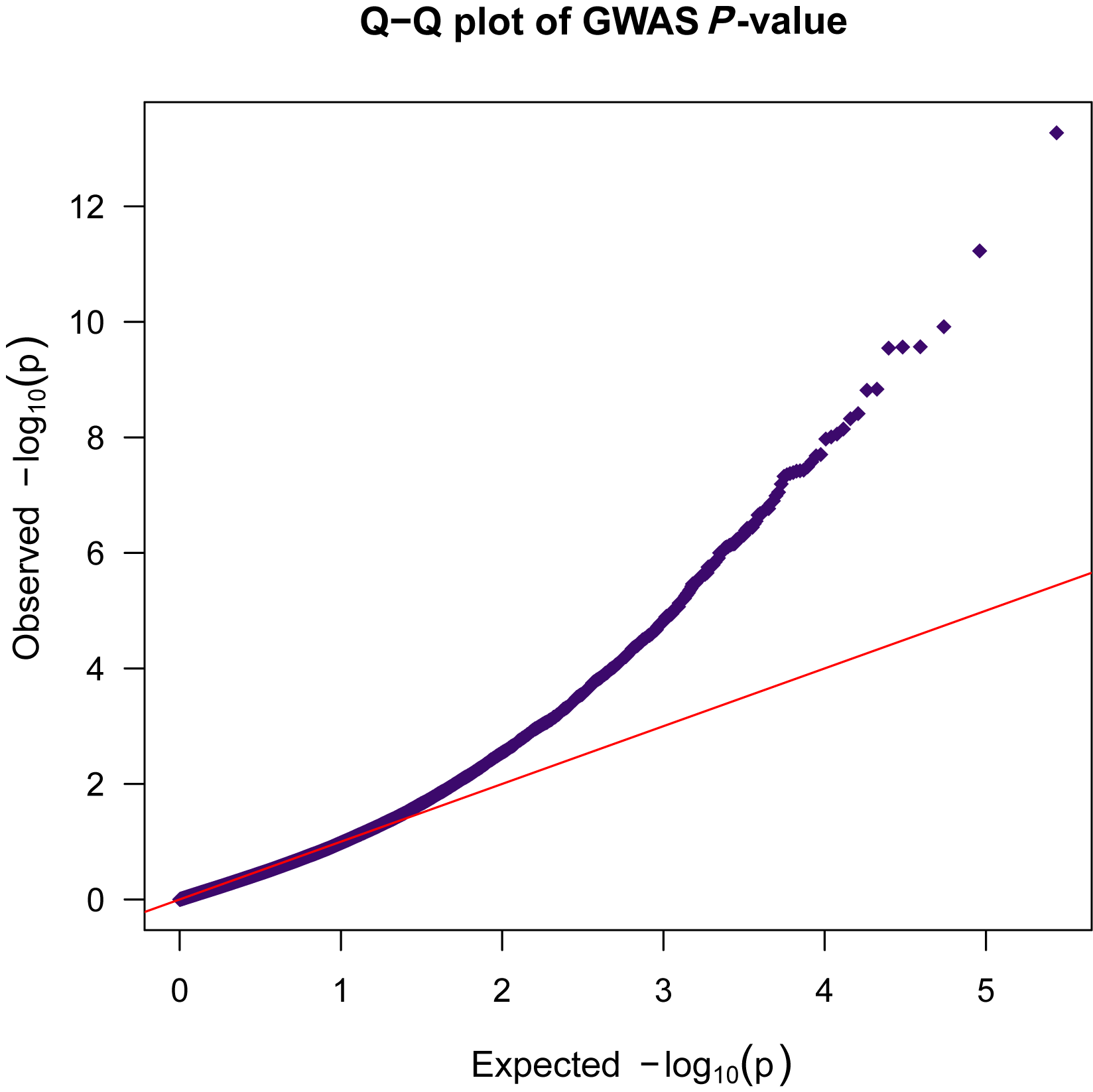


**Figure S18. The Q-Q plot of mitochondrial-nuclear interaction GWAS analysis (p_wald value).**


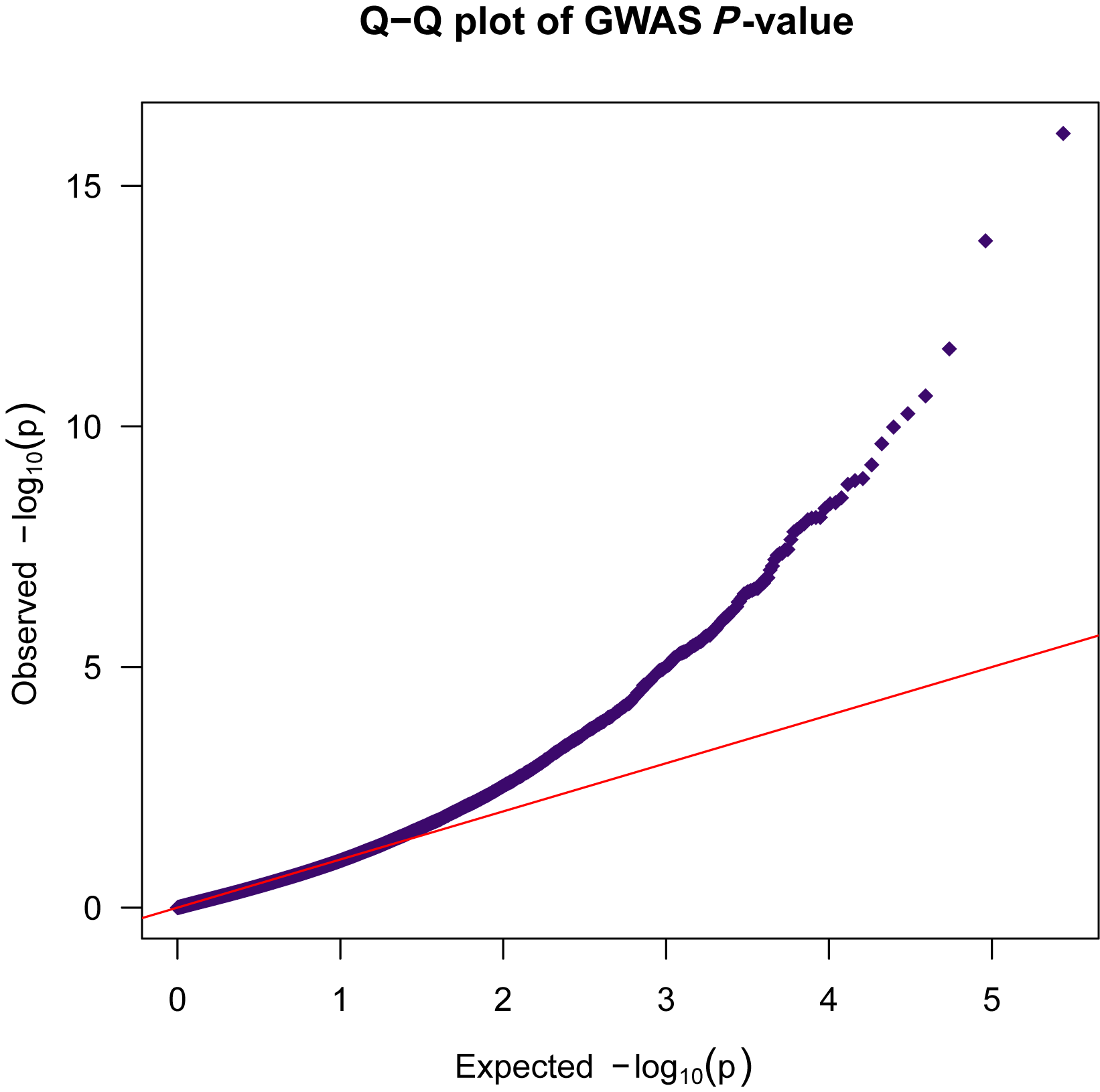


**Figure S19. The Q-Q plot of chloroplast-nuclear interaction GWAS analysis (p_wald value).**


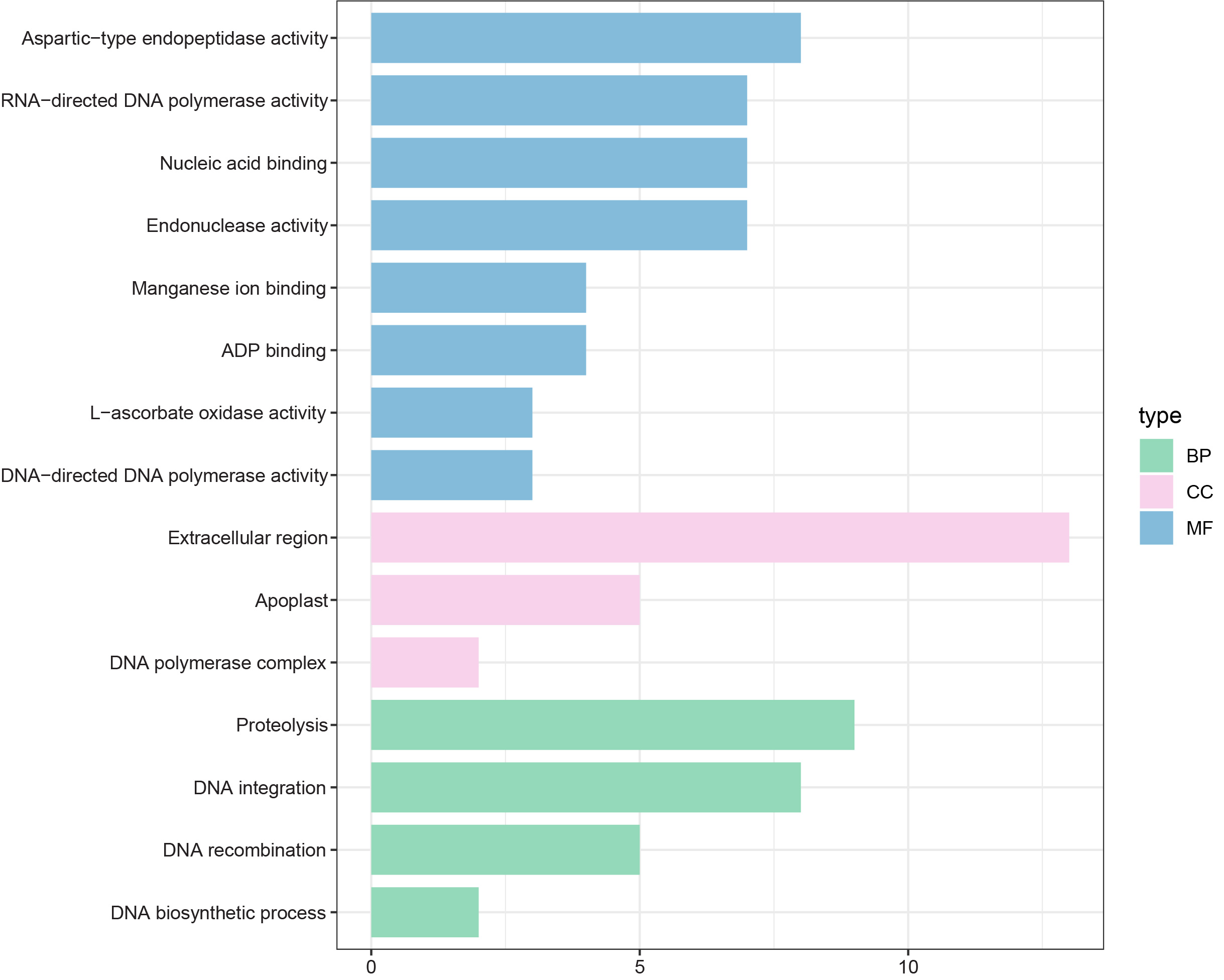


**Figure S20. GO enrichment of mitochondria-nuclear interaction candidate genes.** BP, biological process; CC, cellular component; MF, molecular function.


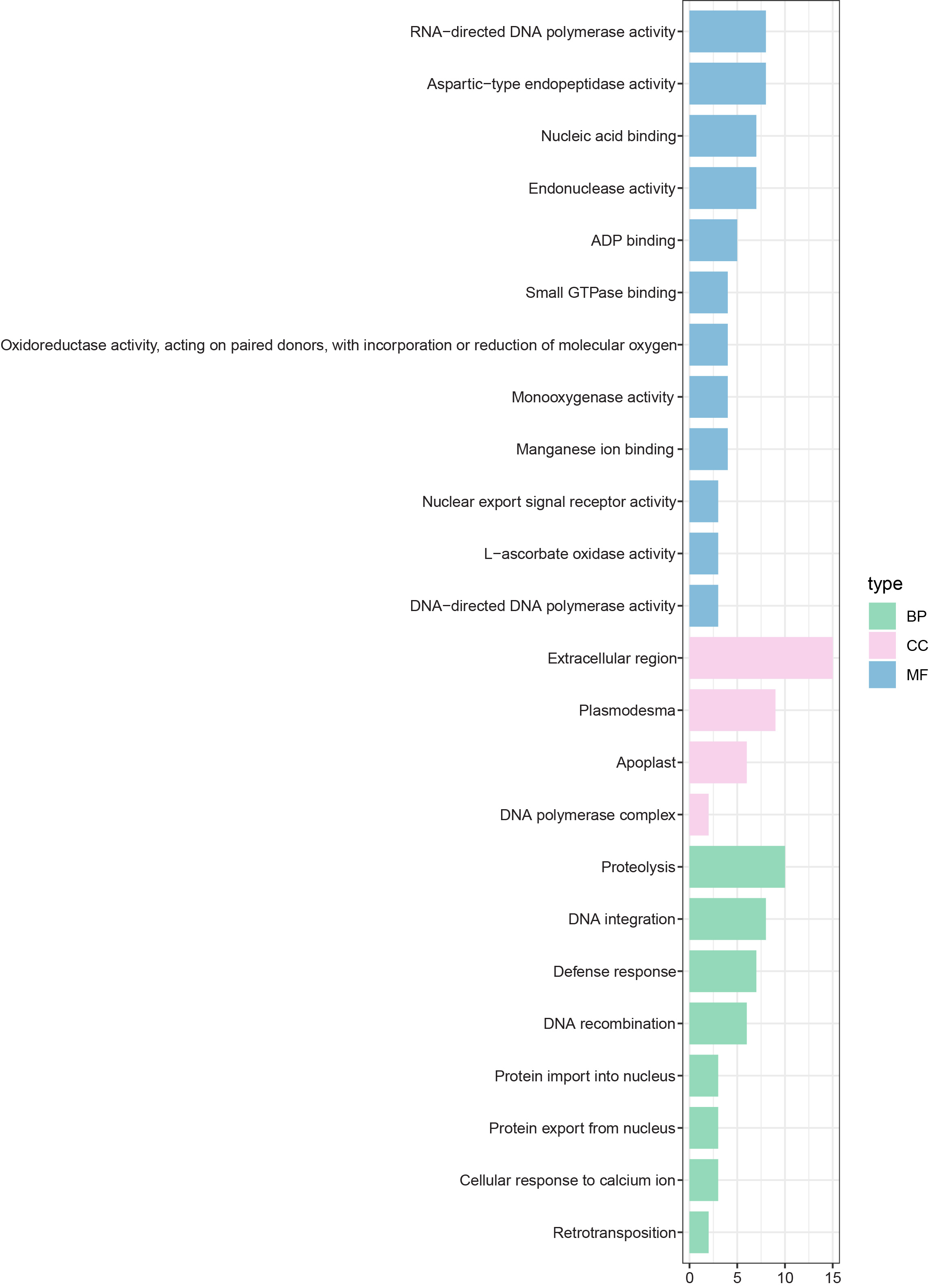


**Figure S21. GO enrichment of chloroplast-nuclear interaction candidate genes.** BP, biological process; CC, cellular component; MF, molecular function.


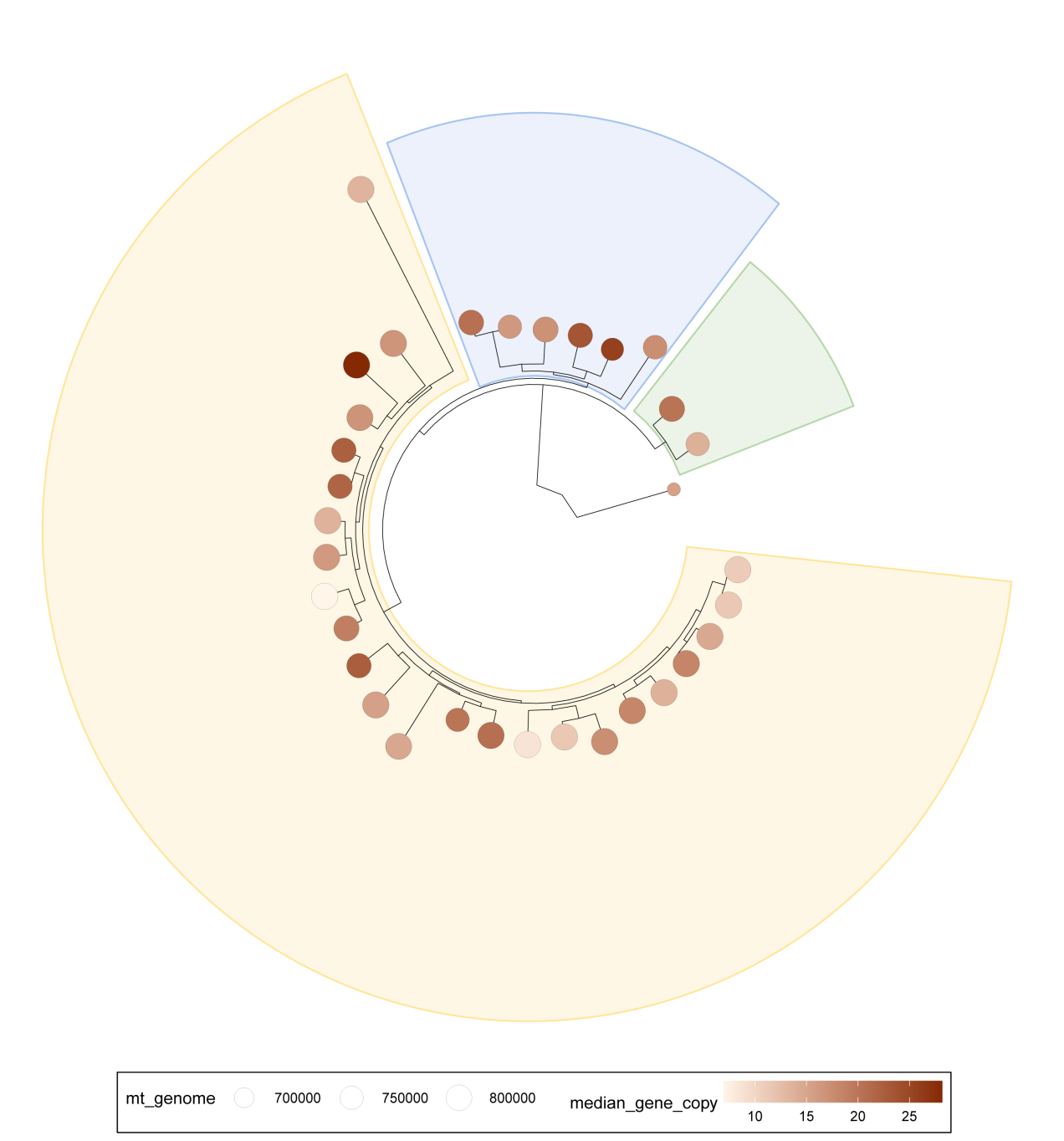


**Figure S22. The grape mitochondrial genome size is related to copy number .** A phylogenetic tree depicting the evolutionary relationships among 33 grape species, with the genome size and copy number indicated by symbols at the tips of the terminal branches. Larger, concentric circles signify greater genome sizes, while darker brown circles denote higher copy numbers. The green sector represents North American wild grapes, the blue sector represents East Asian wild grapes, and the yellow sector represents Eurasian grapes.


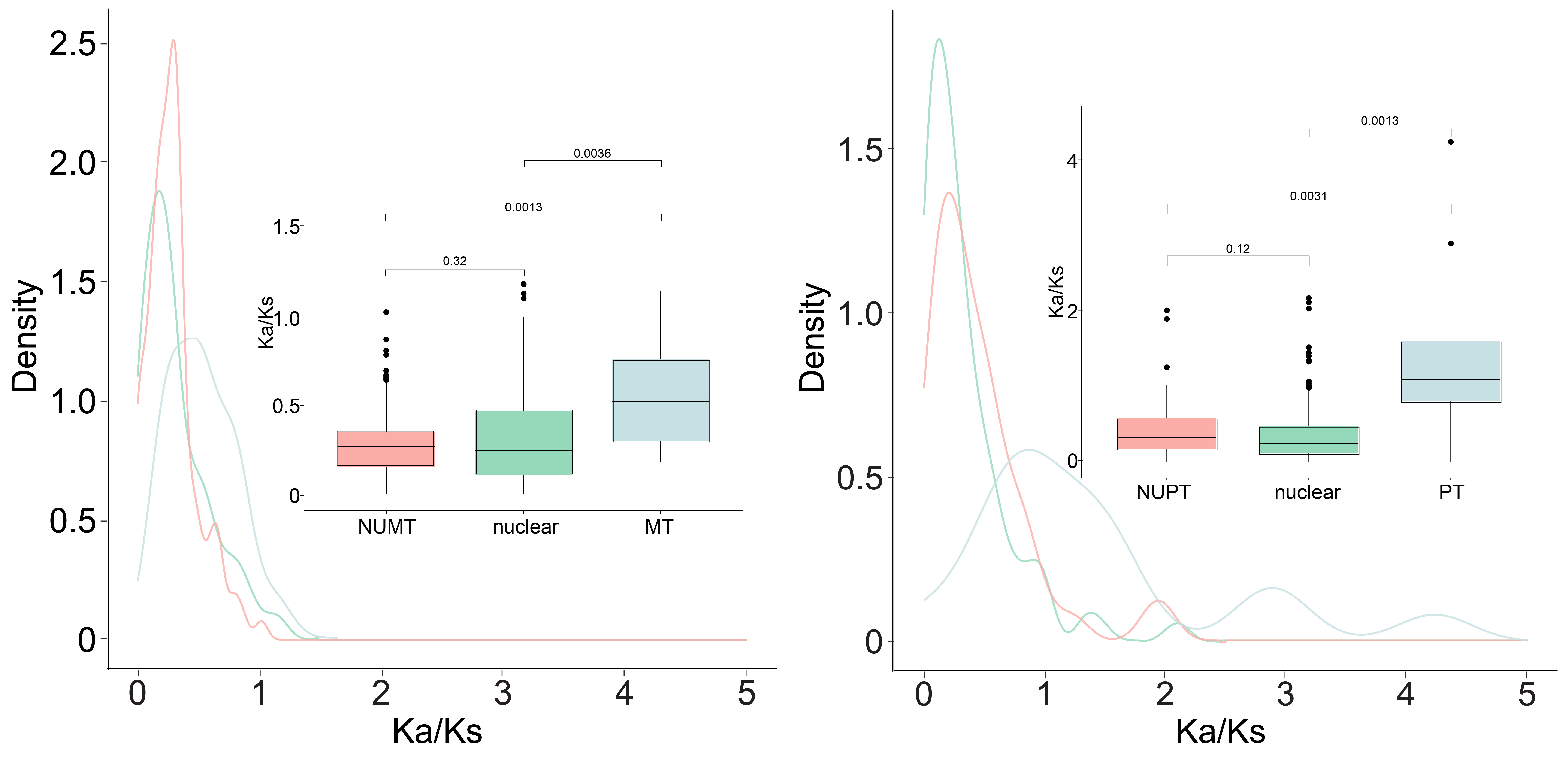


**Figure S23. Comparison of gene evolutionary rates (Ka/Ks) in three groups of wild and cultivated grapes.** To the left of the diagram, the comparison is depicted between the Ka/Ks of the NUMT gene and its adjacent nuclear and mitochondrial genes. Conversely, to the right, the focus shifts to the Ka/Ks comparing the NUPT gene with its surrounding nuclear and chloroplast genes.


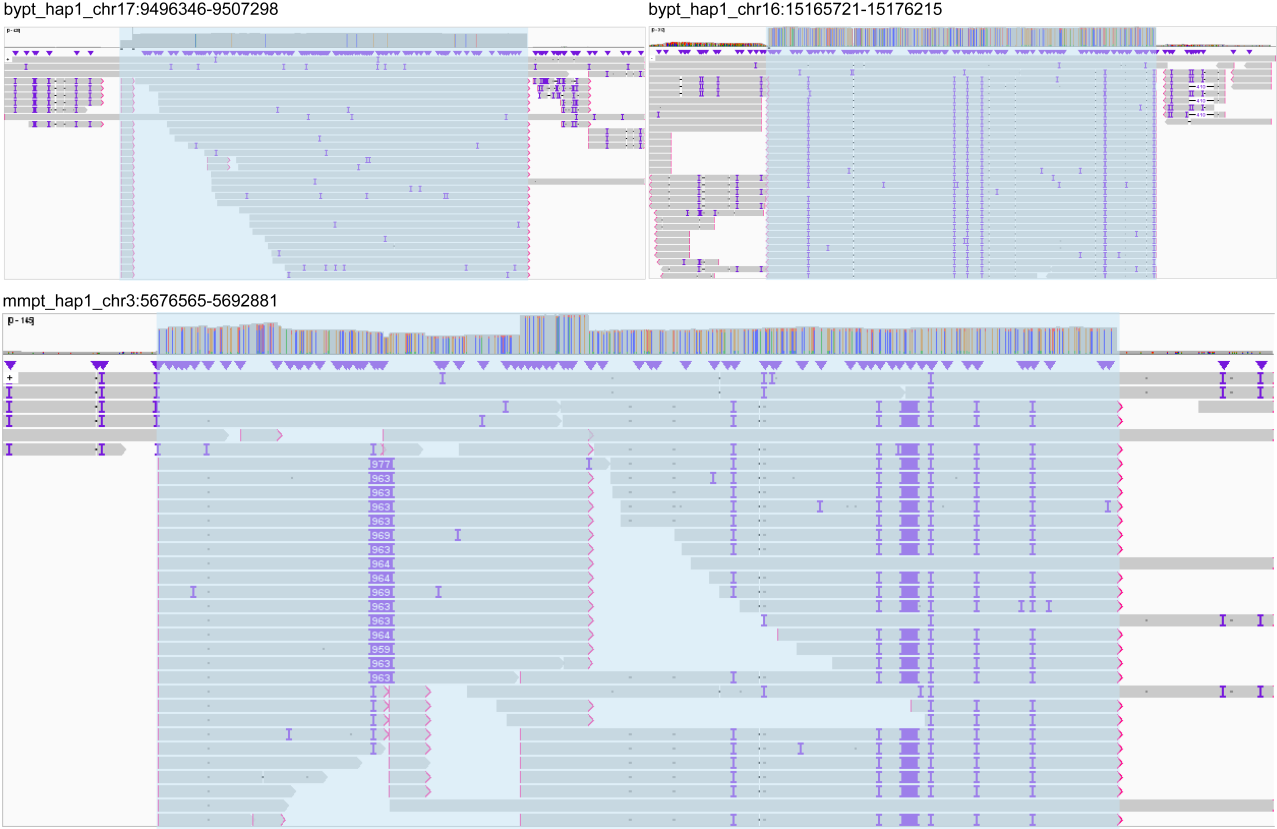


**Figure S24. The IGV plot of NUMTs in grapes.** The region with NUMT fragment reads was highlighted.


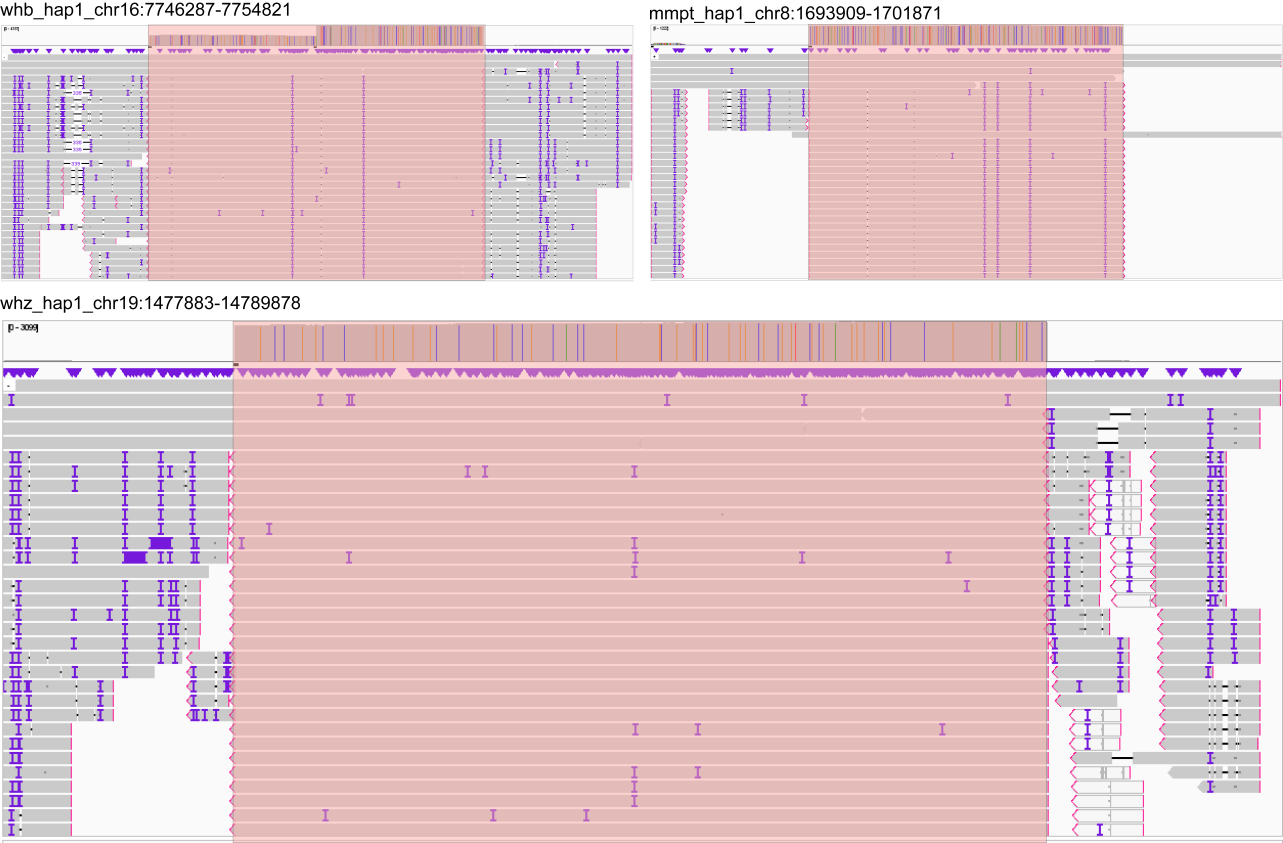


**Figure S25. The IGV plot of NUPTs in grapes.** The region with NUPT fragment reads was highlighted.
